# How can we support children, adolescents and young adults in managing chronic health challenges? A scoping review on the effects of patient education interventions

**DOI:** 10.1111/hex.12906

**Published:** 2019-05-26

**Authors:** Una Stenberg, Mette Haaland‐Øverby, Absera Teshome Koricho, Anne Trollvik, Liv‐Grethe Rajka Kristoffersen, Stine Dybvig, André Vågan

**Affiliations:** ^1^ Norwegian National Advisory Unit on Learning and Mastery in Health Oslo University Hospital Oslo Norway; ^2^ Learning and Coping Center Oslo University Hospital Oslo Norway; ^3^ Institute of Nursing, Faculty of Public Health Inland Norway University of Applied Sciences Elverum Norway; ^4^ Celebral Palsy Young Oslo Norway

**Keywords:** adolescents, children, evaluation, patient education, patient engagement, scoping review

## Abstract

**Objectives:**

This scoping review aims to give a comprehensive and systematic overview of published evaluations and the potential impact of patient education interventions for children, adolescents and young adults who are living with chronic illness and/or impairment loss.

**Methods:**

Relevant literature published between 2008 and 2018 has been comprehensively reviewed, with attention paid to variations in study, intervention and patient characteristics. Arksey and O'Malley's framework for scoping studies guided the review process, and thematic analysis was undertaken to synthesize extracted data.

**Results:**

Of the 7214 titles identified, 69 studies were included in this scoping review. Participant‐reported benefits of the interventions included less distress from symptoms, improved medical adherence and/or less use of medication, and improved knowledge. The majority of studies measuring physical activity and/or physiologic outcomes found beneficial effects. Interventions were also beneficial in terms of decreased use of urgent health care, hospitalization, visits to general practitioner and absence from school. By sharing experiences, participants had learned from each other and attained new insight on how they could manage illness‐related challenges.

**Discussion:**

Study results corroborate previous research suggesting that different types of patient education interventions have a positive impact on children, adolescents and young adults, but research on this field is still in a starting phase. The results summed up in the current review supports the utility of patient education interventions that employ behavioural strategies tailored to the developmental needs of children, adolescents and young adults with different cultural backgrounds.

## 1 INTRODUCTION

Children living with chronic illness are less able to participate in social activities. The daily management of their illness often requires that the whole family adjust to a new way of life.^1^ Adolescents encounter difficulties due to experiences of physical and psychosocial changes, social pressure from their peers and adolescent health‐care transition. Moreover, adolescents tend to be afraid, anxious and shameful of their illness.^2^

Because chronic condition in childhood is one of the major health challenges of this century, gaining skills in self‐management becomes increasingly important.^3^ The challenges are particular worrisome in low‐ and middle‐income countries who experience an increase in the number of young people developing long‐term conditions.^4^

The process where patients are enabled to become actively involved in finding out what is important to them, in making decisions about factors that affect their lives and in taking action to achieve change, is often described as patient engagement.^5^ More recently, the concept of patient engagement has been envisaged as an crucial factor impacting on patients’ ability to self‐manage and as an important goal for medical communication and relationships.^6^ Patient education is a key patient engagement intervention for supporting and enabling children, adolescents and young adults to manage their lives with illness challenges.^7^, ^8^, ^9^ As others have argued,^10^, ^11^ children and adolescents who are living with long‐term health conditions want to gain more knowledge about their illness and its consequences for their everyday life. Many studies also report that young people do not have sufficient knowledge of the transition from child to adult health care.^10^, ^12^

There is a great variety in how patient education interventions are being offered to children, adolescents and young people, and they are often described as complex interventions.^13^ They can be given to groups or to individuals alone, and they can be led by health‐care providers or laypersons.^14^ Group‐based patient education programme, both disease‐specific and general approaches, has been considered an important part of health promotion politics in several Western countries and as being essential for chronic illness self‐management.^15^, ^16^, ^17^

Describing and evaluating the content and impact of how patient education interventions can help to pave the way towards more efficient interventions. A few reviews provide evidence that patient education interventions have been beneficial for children and adolescents with asthma,^2^, ^18^, ^19^ diabetes,^9^, ^20^ cancer,^11^ physical disabilities^3^ or across diagnoses (general paediatric care).^10^, ^12^ However, because of the great variety in type of intervention, setting, design and outcome measure of the included studies, it is not possible to conduct comparative analysis of the results that they present. To date, no review has addressed the full range of studies that have investigated the impact of patient education interventions targeting children, adolescents and young adults. This review aims to give a comprehensive and systematic overview of published evaluations and the potential impact of patient education interventions for children, adolescents and young adults who are living with chronic illness and/or impairment loss.

More specifically, the following questions are addressed:

1. What are the characteristics of the studies, participants and patient education interventions targeting children, adolescents and young people who are living with chronic illness and/or impairment loss as described in the literature?
2. How are patient education interventions designed specifically for children, adolescents and young people evaluated?
3. What impact is associated with patient education interventions targeting children, adolescents and young people, as reported in the literature?

## 2 METHODS

This scoping review is part of a larger research project with the objective to give a comprehensive and systematic overview of published evaluations and the potential impact of patient education interventions for the following:

1. Adults who are participating in group‐based patient education interventions^14^
2. Family members (both adults and children) who are participating in individual or group‐based patient education interventions (in progress)
3. Children, adolescents and young adults who are participating in individual and group‐based patient education interventions (this scoping review)

To capture the health economic aspects, one separate scoping review on the health economic impact of patient education interventions has been conducted and published in 2018.^21^ These four scoping reviews on impact of patient education interventions have followed the same methodological framework^22^, ^23^, ^24^ and are reporting on similar research questions regarding evaluation of patient education interventions targeting different kinds of participants.

As described earlier in the two published reviews^14^, ^21^ in this project, research on the effects of patient education interventions is a relatively new field. To gain a comprehensive overview of the published literature, the research questions were best answered by including different study designs. Thus, scoping review was considered appropriate, also for the current review. Scoping reviews “aim to rapidly identify the key concepts underpinning a research area and the main sources and types of evidence available, and can be undertaken as stand‐alone projects in their own right, especially where an area is complex or has not been reviewed comprehensively before.”^25^ This review followed the five‐stage framework proposed by Arksey and O`Malley^22^ and further refined by Levac et al^23^, ^24^

The following specifications were considered relevant for this scoping review:

- Population: target population includes children, adolescents or young adults between the age of 0 and 25 who are living and coping with any type of chronic illness and/or impairment loss.
- Intervention: any kind of face‐to‐face patient education intervention aimed at supporting self‐management, and optimizing health and well‐being, led by health‐care professionals and/or lay participants.
- Comparisons: usual care/treatment, different types of interventions or no comparisons.
- Outcomes: any of a range of different types of impacts and outcomes related to social, health, psychological, health economic or behavioural aspects.

We have conducted systematic searches in the following electronic databases from 01 January 2008 to 01 February 2018: MEDLINE, EMBASE, PsychINFO, AMED, CINAHL, SweMed+, ERIC and Cochrane Library Online. The literature searches have followed the PICO principles combined with and “OR” within group and subsequently combined with an “AND” between groups. We have used a wide variety of terms in the database thesaurus in addition to free text/key word method:

- Participants: children, adolescent, youth, paediatric, young people, young adults.
- Intervention: self‐management programme/education/group, group support programme, learning and mastery course, patient education, patient education course/programme/intervention, patient engagement, peer support, group intervention, group‐based education/programme.
- Diagnosis/health: chronic disease, chronic illness, lung diseases, asthma, pain, fatigue syndrome, irritable bowel syndrome, gastrointestinal, osteoporosis, HIV infections, arthritis, diabetes mellitus, hypertension, myocardial ischaemia, heart failure, stroke, neoplasms, fibromyalgia, mental disorders, cardiovascular disease, obesity, COPD, lung illness, cancer.

We only included studies published in English, Norwegian, Swedish or Danish in peer‐reviewed journals. The studies were required to have investigated: the impact or effects over time (a) of individual‐ and/or group‐based patient education interventions (b) for children, adolescents and/or young adults living with any type of chronic illness challenges (c). Interventions based mainly on the use of technology were excluded. A different search strategy would have been required in order to capture the full scope of such studies.

**Figure 1:**
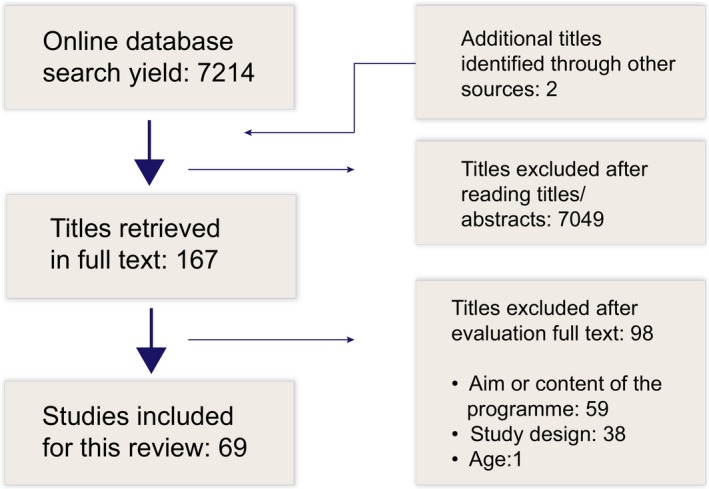
Included and excluded studies

All the members of the study group were involved in the discussions of the search strategy, and our discussions helped clarify the inclusion and exclusion criteria for this review. A broad search in all the relevant databases was conducted, with no restrictions. The search of the online databases yielded 7216 articles, and 7049 of these articles were excluded because they did not meet the inclusion criteria (Figure 1). The remaining 167 articles were obtained in full text and read by the first author and one co‐author. Of these, 98 articles were excluded, because inclusion criteria were not met. There were few disagreements about article inclusion, and these were resolved by discussion in the study group to reach consensus. As is frequently seen in research on patient education interventions tailored to adult patients,^14^ the interventions for children, adolescents and young people were often poorly described. In addition, interventions with similar‐sounding names could be very different in content. Therefore, every intervention was screened before inclusion, and 59 were excluded because the aim or content of the patient education intervention did not meet the criteria. A final total of 69 articles were included for analysis in this review.

Information about study characteristics, participant characteristics, descriptions of interventions, methods and results was collected on data extraction forms and reported separately for each study in an evidence summary Table S1 (Supporting Information).

The research included in this study showed significant variation in type of intervention, design and outcome measures. The 69 study results comprising the research material under scrutiny were compared according to the type of patient education intervention, diagnosis and type of outcome measured in order to find patterns and similarities. The data summarization was mainly done by two of the authors (US and MH) and subsequently validated by all co‐authors.

## 3 RESULTS

In this scoping review, 69 research‐based studies have been included. The presentation of the results is organized according to the main questions addressed in this review.

### 3.1 Characteristics of the studies

The studies were published between 2008 and 2018. Most of the studies (47/69) were published in 2012 or later. The 69 published studies were conducted in 26 different countries (Table 1).

**Table 1:** Country and number of studies

Country	Number of studies
United States of America	28
Australia	7
Turkey	4
United Kingdom	3
Canada	2
Norway	2
Taiwan	2
Germany	2
China	2
Jordan	1
Sweden	1
Australia/Jordan	1
Chile	1
Netherlands	1
Poland	1
Switzerland	1
Iran	1
Italy	1
Belgium	1
France	1
Mexico	1
Pakistan	1
Denmark	1
Thailand	1
Serbia	1
Brazil	1
Total	69

Among the 69 research‐based studies, three of them employed qualitative designs, and two made use of mixed‐method designs. Of the quantitative studies, 43 studies were randomized controlled trials with experimental design, and 21 had an observational analytical design (cohort or case‐control studies). Fifty of the quantitative studies (50/66; 76%) compared the outcomes of patients participating in patient education interventions with those of a control group of patients or compared outcomes of participation in different patient education interventions. In most of these studies, participants in control groups received usual care and treatment. All the quantitative studies reported changes over time, before and after participating in a patient education intervention:

- Before and immediately after the intervention: 4 studies
- Before and 12 months after intervention: 54 studies
- Before and between 1 and 2 years after intervention: 6 studies
- Before and more than two years after the intervention: 2 studies

### 3.2 Participant characteristics

A total of 15 124 participants were included in the studies for this scoping review (Table 2). The mean age of children and adolescents was 12.1 years (17 studies did not report mean age); 31/69 studies reported ethnicity, with the mean of 46.5% white participants.

**Table 2:** Study participants: gender and age

	Number of participants (%)
Total sample	15.124 (100%)
Gender	
Women	7.160 (47.34%)
Men	6869 (45.42%)
Not reported	975 (6.45%)
Age	
Mean age, y	12.14
Range, y	3 to 29

### 3.3 Classification of chronic condition

A breakdown of the 69 studies by chronic condition is provided in Table 3. The largest number of studies included in this review focused on asthma (30/69), followed by diabetes (15/69).

**Table 3:** Diagnosis and number of studies

Diagnosis	Number of studies
Asthma	30
Diabetes	15
Obesity	5
Mental health disorders	4
Chronic illness (various)	4
Cancer	3
Chronic fatigue syndrome	2
Pain	1
Organ transplanted	1
Oesophageal atresia	1
Autism spectrum disorder	1
Migraine	1
Total	69

### 3.4 Characteristics of the patient education interventions

The interventions had diverse origins, aims, target groups, settings and number of modules and were delivered by different health‐care personnel and/or peers. This is described in detail in Supporting Information Table S1. The interventions in these studies were face‐to‐face patient engagement interventions aimed at helping children, adolescents, young adults and families develop coping skills and gain knowledge to manage illness, health and everyday life.

In 13 of the studies, the interventions were tailored to the participants’ developmental phase, educational level or to ensure cultural comparability. Four (5.8%) of the studies were focused on the transition from child‐ to adult‐oriented health‐care services. A majority of the interventions concerned conventional self‐management education with pathophysiology and information, medication and action planning, lifestyle guidelines, self‐care, symptom management and adherence, but mostly in addition to other components such as problem‐solving (23/69; 333%), planning (51/69; 73.9%) and practising coping skills (37/69; 53.6%). Twenty‐one of the interventions were supplemented with written or multimedia material (21/69; 30.4%) and/or entertaining materials (8/69; 11.6%), phone calls (4/69; 5.8%) or website (3/69; 4.3%).

Commonly, interventions were led by trained facilitators (28/69; 40.6%) or multidisciplinary teams (19/69; 26%). Eight interventions (8/69) were led by nurses, and seven (7/69) were delivered by a nurse and a therapist in collaboration. Other personnel reported to be involved were family health coaches (3/69), community health‐care providers (3/69), physiotherapists (2/69), clinicians (1/69), a team of general practitioners and experts in physical activity (1/69), educators and dietitians (1/69) and study team counsellors (1/69). In two cases, a health‐care team, fellow peers and school personnel were responsible for implementing the intervention (2/69). Two of the interventions were peer‐led, and three studies provided no information on which personnel delivered the interventions.

The duration of the interventions ranged from one session (12/69; 17.4%), to two to eight sessions (33/69; 47.8%), to 10 sessions or more (5/69; 7.2%). Session lasted anywhere from 15 minutes to 2.5 hours in different studies. Seven interventions lasted between two and five whole days (7/69; 10.1%). One study compared 13 cognitive behavioural therapy sessions with four psychoeducational sessions. Four studies (4/69; 5.6%) reported no information on number of sessions, but a duration from 6 to 12 months. One study provided no information about the duration of the intervention.

Interventions for individual patients comprised 35/69 (50.7%), whereas 22 included family or support persons, and five combined joint and separate sessions. Among the group‐based interventions (28/69; 40.6%), nine included family, four had separate groups for the caretakers, and one included both family and teacher. Two of the studies (2/69; 2.9%) combined groups and individual sessions, and three studies (3/69; 4.3%) did not report the mode of the intervention.

The interventions were offered in hospitals (50/69; 72.5%), schools (10/69; 14.5%) or were home‐based (9/69;13%). Seven (7/24; 10.1%) interventions were offered in general practice, community centres, university training centres and primary care. Two (2.9%) studies lacked a description of the setting in which the intervention was delivered.

### 3.5 Characteristics of methods for evaluation

The studies included in this review have used a wide range of different outcome measures. Outcomes concerning disease management and coping, knowledge about conditions and treatments, symptom severity, self‐efficacy, self‐management behaviours, empowerment, self‐esteem and health economy were frequently measured. The health economic evaluations were measured in terms of hospitalization, use of urgent and preventive health services and number of days absent from school/college or work. Table 4 presents all the validated outcome measures and gives references to the primary source and/or validation studies. The table also shows whether the outcome measure is typically associated with a specific diagnose or is used across diagnoses.

**Table 4:** Outcome measures used in the research‐based studies included in this scoping review

Outcome	Condition	Outcome measure
Disease	Asthma	Self‐Administered Nicotine Dependence Scale^99^
management and	Asthma	Asthma Control Test (ACT)^100^, ^101^
coping	Asthma	Childhood Asthma Control Questionnaire (ACQ)^102^
	Asthma	The Asthma Control Questionnaire^103^
	Asthma	Child Asthma Control Test (CACT)^104^
	Asthma	Asthma Inventory for Children (AIC)^105^
	Asthma	The Asthma Belief Scale^106^
	Cancer	Pediatric Cancer Coping Scale (PCCS)^107^
	Diabetes	Summary of Diabetes Self‐Care Activities^108^
	Diabetes	Issues in Coping with T1D‐Child Scale^109^
	Across conditions	Kid Cope^110^
Knowledge	Asthma	Asthma Knowledge Consumer Questionnaire (CQ)^111^
	Asthma	Asthma Knowledge Test^112^, ^113^
	Asthma	Questions About Asthma Questionnaire^114^
	Autism	Autism Knowledge Quiz (AKQ)^77^
	Diabetes	Diabetes Knowledge Test^115^
Self‐esteem	Across conditions	The Rosenberg Self‐Esteem Scale (RSES)^116^, ^117^
Physiological	Asthma	Peak expiratory flow rate (PEF)
	Across conditions	Waist circumference (WC)
	Across conditions	Hip circumference (HC)
	Across conditions	BMI score
Health‐related quality of life	Asthma	Pediatric Asthma Quality of Life Questionnaire (PAQLQ)^118^
	Asthma	Mini Asthma Quality of Life Questionnaire (Mini‐AQLQ)^119^
	Diabetes	Diabetes Quality of Life Scale (DQOLY‐SF)^120^
	Across conditions	Medical Outcomes Survey‐Short form (SF‐36)^121^
	Across conditions	EuroQol Questionnaire (EQ‐5D)^122^
	Across conditions	Health‐related Quality of Life (DISABKIDS)^123^
	Across conditions	Quality of Life Questionnaire (DUCATQOL/DUX‐25)^124^
	Across conditions	The Pediatric Quality of Life Inventory (PedsQL)^125^
	Across conditions	EUROHIS QOL‐8^126^
Psychosocial	Diabetes	Diabetes Family Responsibility Questionnaire (DFRQ)^127^
	Diabetes	Diabetes Family Conflict Scale (DFCS)^128^
	Across conditions	Communal Family Mastery Scale^129^
	Across conditions	Transition Readiness Assessment Questionnaire (TRAQ)^130^
	Across conditions	Perceived Stress Scale^131^
	Across conditions	Strengths and Difficulties Questionnaire (SDQ)^132^
	Across conditions	University of California Los Angeles Loneliness Scale (UCLA)^133^
Health economics	Across conditions	Quality Adjusted Life‐Years (QALYs)^134^
	Across conditions	The Health Utilities Index Mark 2 (HUI‐2)^135^
	Across conditions	The Child Health Utility 9D (CHU9D)^136^
	Across conditions	Number of hospital days
	Across conditions	Number of re‐hospitalizations
	Across conditions	Number of Emergency Department visits
	Across conditions	Number of sick days
	Across conditions	Number of doctor visits
	Across conditions	Number of preventive visits
	Across conditions	Number of urgent care visits
	Across conditions	Number of missed schooldays
Self‐efficacy/empowerment	Asthma	Asthmatic Child and Adolescent Self‐Efficacy Scale (ACASES)^137^
	Asthma	Child Asthma Self‐Efficacy Questionnaire^138^
	Diabetes	Self‐Efficacy for Diabetes Scale^139^
	Diabetes	Diabetes Empowerment Scale‐Short Form^140^
	Across conditions	General Perceived Self‐Efficacy Scale (GSE)^141^
Impact of illness/symptom	Asthma	Number of asthma symptom free days
	Diabetes	The Diabetes Behavior Rating Scale^142^
	Across conditions	Hamilton Depression Rating Scale (HDRS)^143^
	Across conditions	Children's Depression Inventory (CDI)^144^
	Across conditions	Young Mania Rating Scale (YMRS)^145^
	Across conditions	Chalder Fatigue Questionnaire^82^
	Across conditions	Pittsburgh Sleep Quality Index (PSQI)^146^
	Across conditions	Modified Yale Preoperative Anxiety Scale (m‐YPAS)^147^
	Across conditions	Beck Depression Inventory (BDI)^90^
	Across conditions	Depression Anxiety Stress Scale—short version (DASS‐21)^148^
	Across conditions	Centers for Epidemiologic Studies Depression Scale (CES‐D)^149^, ^150^
	Across conditions	Patient Health Questionnaire^151^
Self‐Management	Across conditions	Work and Social Adjustment Scale^152^
	Across conditions	Patient Activation Measure (PAM)^153^
Global improvement and satisfaction	Across conditions	Global Assessment of Functioning (GAF)^154^
	Across conditions	Health of Our Nation Outcome Scale (HoNOS)^155^
	Across conditions	Number of sessions attended
	Across conditions	Satisfaction With Life Scale^156^
	Across conditions	Arizona Integrative Outcomes Scale‐24 and Arizona Integrative Outcomes Scale‐30^157^
Physical activity/nutrition	Across conditions	Self‐reported physical activity levels (IPAQ‐SF)^158^
	Across conditions	Physical Activity Scale (PAS)^159^
	Across conditions	Block Food Frequency Questionnaire (FFQ)^160^
Medication Adherence	Across conditions	Medication Adherence Rating Scale (MARS)^161^
Mindfulness	Across conditions	Avoidance and Fusion Questionnaire for Youth (AFQ‐Y8 short version)^162^
		Children's Acceptance and Mindfulness Measure (CAMM short version)^163^
Resiliency	Across conditions	Resiliency Scales for Children and Adolescents (RSCA)^164^

### 3.6 Psychosocial outcomes

One or several psychosocial outcomes had been investigated in most of the studies. Symptom management, medical adherence and/or medication use, health‐related quality of life and knowledge were frequently measured. *Management* or frequency of symptoms was examined in 44 studies. Four of the studies that had investigated symptom frequency found no differences,^26^, ^27^, ^28^, ^29^ and 25 studies reported reduction of symptoms and/or awareness of symptom triggers.^30^, ^31^, ^32^, ^33^, ^34^, ^35^, ^36^, ^37^, ^38^, ^39^, ^40^, ^41^, ^42^, ^43^, ^44^, ^45^, ^46^, ^47^, ^48^, ^49^, ^50^, ^51^, ^52^, ^53^, ^54^ Ten studies from interventions tailored to children and adolescents with asthma reported improved *asthma control* 51, 55, 56, 57, 58, 59, 60 and decreased number of *asthma exacerbations.* 38, 55, 56, 61, 62 One study found no changes in asthma control.^57^

All the studies that had investigated *medical adherence and/or use of medication* reported better adherence to medications^31^, ^32^, ^38^, ^55^, ^63^, ^64^, ^65^ and/or decreased need for use of medication.^53^, ^55^, ^65^, ^66^, ^67^, ^68^ *Health‐related quality of life* was measured in 23 studies. From these, eleven did not find any significant differences between the intervention group and the control group,^26^, ^27^, ^29^, ^57^, ^63^, ^68^, ^69^, ^70^, ^71^, ^72^, ^73^ while 12 studies found significant effects or improvements in intervention groups over time.^40^, ^42^, ^52^, ^53^, ^55^, ^59^, ^60^, ^66^, ^74^, ^75^, ^76^

*Knowledge* was investigated in 10 studies.^41^, ^49^, ^52^, ^57^, ^58^, ^65^, ^69^, ^74^, ^77^, ^78^, ^79^ All these studies reported significant improvements in knowledge scores after intervention. Eight studies had investigated changes in *self‐efficacy*. Three of these found significantly greater self‐efficacy in intervention participants^38^, ^74^, ^75^; in addition, two studies found improvements, albeit not significant,^52^, ^58^ and three studies found no effects on self‐efficacy.^39^, ^76^, ^80^ Whereas self‐efficacy describes persons’ belief in their capacity to arrange and carry out a course of action,^81^ empowerment is a consequence of achieving self‐efficacy. Only one study had *empowerment* as one of several outcome measures, and this study found no changes in empowerment.^49^

Four studies measured *strengths and difficulties* by using a behavioural screening tool for psychopathology and adaptation. One study found that, when compared to the control group, participants in the intervention group became more aware of their strengths as well as the difficulties connected to their diagnosis.^77^ One study was unfortunately underpowered and found no differences.^73^ One of the two studies that compared cognitive behavioural therapy‐based intervention with psycho‐education failed to find significant differences between the groups.^82^ The second study, however, found significant changes in the cognitive behavioural therapy group controlled for baseline scores.^44^

*Self‐esteem* was investigated in three studies. Two of these studies found increased self‐esteem after participation in group programmes for adolescents with type 1 diabetes^78^ or mental health challenges.^83^ However, the third study, investigating a one‐on‐one intervention for young people diagnosed with psychosis, did not detect statistically significant changes.^35^ Two studies had measured the level of *patient engagement* in their health. The interventions investigated in these studies did not affect activation over time.^69^, ^72^ One study had measured differences in *social support* and found that intervention participants gained significantly more social support when compared to participants receiving ordinary care.^78^ One study investigated *mindfulness* changes and found significant higher mindfulness scores among adolescents in the intervention group compared to those in the control group.^83^

### 3.7 Physical activity and physiologic outcomes

A variety of physical activity and physiologic outcomes were measured in 23 studies. Of these, 19 studies found improvements, 29, 30, 33, 35, 39, 41, 43, 49, 55, 71, 75, 78, 84, 85, 86, 87 and four studies found no changes.^63^, ^70^, ^76^, ^88^ Differences in blood glucose control were measured in 15 studies. Of these, four studies found no differences,^63^, ^70^, ^76^, ^88^ while 11 studies found improvements in glycaemic control.^29^, ^39^, ^41^, ^43^, ^49^, ^50^, ^78^, ^84^, ^86^, ^87^, ^89^ Improvements in physical activity or greater adherence to behavioural support were reported in all studies that had measured physical activity.^33^, ^35^, ^44^, ^71^, ^75^, ^90^ Nine studies had measured body mass index (BMI) scores. Of these, eight found greater reductions in BMI in the intervention group than controls.^30^, ^35^, ^41^, ^43^, ^46^, ^50^, ^84^, ^85^ One study found no impact on BMI.^76^

### 3.8 Health‐care utilization

Of the studies dealing with patient education interventions, 14/17 studies resulted in beneficial effects as measured by one or several health economic outcomes.^31^, ^33^, ^38^, ^42^, ^45^, ^49^, ^53^, ^55^, ^56^, ^59^, ^61^, ^64^, ^66^, ^68^ Three studies found no health economic impact or effects of the interventions.^62^, ^91^, ^92^ Use of urgent health care or the frequency of emergency visits, visits to the local doctor and hospitalization, and missed schooldays were frequently investigated in these studies. Use of urgent health care was investigated in 13 studies, and 11 of these found significantly decreased use of urgent health care or trends towards beneficial effects,^31^, ^33^, ^38^, ^42^, ^45^, ^49^, ^55^, ^59^, ^64^, ^68^ while two studies found no effects.^91^, ^92^ Of the six studies that had measured hospitalization, three found beneficial effects,^31^, ^45^, ^61^ while three found no effects.^53^, ^56^, ^92^ Six studies found beneficial effects: two in terms of fewer visits to general practitioners^59^, ^66^ and four in terms of less absence from school.^38^, ^56^, ^66^, ^68^ Apart from one study involving children with diabetes,^49^ all the patient education interventions in the studies that had investigated health‐care utilization were targeting children and adolescents with asthma.

### 3.9 Perceptions of participation—results from qualitative studies

The five studies with a qualitative approach had explored how children and adolescents who were living with diabetes, asthma, stress‐related problems or cancer experienced participating in patient education interventions.^1^, ^40^, ^54^, ^93^, ^94^ Overall, the studies showed that by sharing experiences, participants had learned from each other and attained new insight. They also learned through interaction with educational material and from health‐care personnel. Adolescents in one study described that the most challenging part was deciding to sign up for the course because it required them to admit to themselves that they needed the course.^94^ The studies showed that sharing experiences with peers spurred meaningful learning experiences and an empowering process. Children and adolescents found it easier to talk about their diseases and to share their thoughts and feelings about living with health challenges with family members and others after participating in patient education interventions.^1^, ^54^, ^93^, ^94^ In general, children and adolescents experienced alleviation, comfort and a feeling of hope when realizing that there are others struggling with the same issues.^1^, ^94^

And that’s what it’s about, meeting in a group: where everyone has the same problem, then everybody dares to raise issues. That’s really great. Nobody judges anybody, no way. ^94^ p. 8

Another important result from these five studies was that participants gained insight and concrete knowledge about the disease, its symptoms and potential causes, and how they could manage all the daily illness‐related challenges. For example, they learned how to manage symptoms and to be more aware of triggers and stress responses, how to take medication and cope with side‐effects, or other difficult situations and problems. Adolescents taking part in a stress management course found it useful to learn and understand how physical discomfort is highly related to stress in daily life.^94^ Participants in all five studies had learned concrete problem‐solving skills. How these skills and knowledge could be used in everyday life and activities was verbalized in the groups. Despite the perceived benefits of participating, after completing the courses some found it hard to remember what they learned, making it difficult to sustain a change in behaviour over a long period of time. These participants suggested including re‐education as an additional component in forthcoming courses, also because certain topics would become more significant when they got older.^93^

## 4 DISCUSSION

### 4.1 Summary of main findings

This scoping review is based on 69 studies published between 2008 and 2018. The major amount of the included studies was conducted in North America, Australia and Europe and had an experimental or observational analytical design and reported changes over the first year after intervention. Only eight studies had evaluated changes after more than one year after intervention. A total of 15 124 children, adolescents and young adults were included as participants in these studies, approximately equal numbers of males and females, with a mean age of 12.1 years. Most of the interventions were diagnosis‐specific (the main diagnoses are asthma, diabetes, obesity and cancer), and only four interventions included participants across different diagnoses.

As described by Lorig and Holman (2003),^8^ patient education aims to enable patients to understand the process of their illness, to acquire knowledge and skills to manage medical and disease challenges, to adjust treatment of their condition and to maintain quality of life. The patient education interventions in this review, both individual and group‐based, were aimed at helping children, adolescents, young adults and families develop coping skills and gain knowledge to manage illness, health and everyday life. The interventions were led by trained facilitators and multidisciplinary teams and were offered in hospitals and schools.

Participants considered the interventions beneficial, reporting less symptom distress, improved medical adherence and/or less use of medication, and improved knowledge. Several studies employed health‐related quality‐of‐life measurements, but only 12/23 studies found significant effects or improvements. The qualitative studies showed that by sharing experiences, children and young people learned from each other and created new insight, for example knowledge about disease, symptoms and potential causes, and how they could manage all the daily illness‐related challenges. Physical activity and physiologic outcomes were investigated in 24 studies, and 20 of these found beneficial effects on blood glucose control, physical activity and BMI scores.

In this review, 14/17 studies found that participation in patient education interventions was beneficial in terms of decreased use of urgent health care, hospitalization, visits to general practitioner and fewer missed days from school. No studies documented that participation in patient education interventions had any unintended negative effects on children, adolescents or young adults.

### 4.2 Strengths and limitations

This study shares the limitations that are inherent to scoping reviews in general.^14^, ^21^ Balancing breadth and depth of analysis is challenging, and a further complication lies in synthesizing studies with different designs and methods in the same review.^95^, ^96^ The motivation for doing this scoping review was to provide insight into systematic evaluations of patient education interventions in health care for children, adolescents and young adults who are coping with long‐term illness challenges. Our aim was to get an overview and capture the whole breadth of studies that had evaluated these types of interventions. Therefore, none of the studies have been excluded on the basis of methodological characteristics. In line with scoping review recommendations,^97^, ^98^ we have not performed assessments of methodological limitations of the studies. The purpose of scoping reviews is to give an overview of the literature on a certain topic and normally to not include evaluations of methodological weaknesses or risk of bias.

In this review, we have included studies on patient education interventions for children, adolescents and young adults with any type of long‐term illness challenges. We wanted to capture as many relevant studies as possible; therefore, we used a large number of synonyms in our searches in the databases. Nonetheless, the list of search terms was neither complete nor exhaustive. Since we had a broad definition of patient education interventions, we could include a wide range of interventions. Similar to our earlier reviews on patient education,^13^, ^20^ the interventions in this review also varied in terms of setting, theoretical basis, target groups, modules, duration and personnel/lay participants. In addition, the components of “ordinary care” or “waitlist controls” were often not described.

Chronic illness in children affects daily functioning in the whole family. Some of the interventions in the included studies involved parents, but parental outcomes are not included in this review. Another limitation we also found in our previous reviews on patient education^14^, ^21^ is the lack of information about the relationship between demographic characteristics and reported outcomes. Since the largest share of these studies has been conducted in USA and Australia, much of what we know is based on people with Western ethnicity. We are fully aware that the success of any patient education intervention in general is likely to be determined by local factors and situations, which are often difficult to model and replicate. Therefore, the general transferability of the results from the included studies in this scoping review and applicability to clinical practice has not been specifically analysed.

It is important to be aware of that the proportion of the included studies reporting significant effects of patient education interventions may be inflated due to publication bias. Finally, since this scoping review aimed to give breadth and comprehensiveness, it was necessary to compromise and reduce the depth of analysis and validity assessment.

### 4.3 How and why it agrees or disagrees with the existing literature

Results from earlier reviews are supported by the results from this study and indicate that patient education interventions have positive effects, reducing the frequency of hospitalizations and emergency visits,^18^ improving self‐management of chronic illness, the self‐efficacy of young people with long‐term conditions and the quality of life^2^, ^19^ of children with asthma. One review of the structured, more behaviourally focused programmes for youths with diabetes demonstrate beneficial results on young people's ability to manage their emotions, level of parent–adolescent conflict, adherence to medical treatment and sometimes metabolic control.^9^ Two reviews suggest that interventions facilitate a better transfer from paediatric to adult health care.^10^, ^12^ Nevertheless, due to the great variety in interventions and inclusion criteria, a comparison of the results presented in the reviews is not possible. To the best of our knowledge, no previous review has included and summed up such a broad scope of studies, including evaluations of benefits from patient education interventions for children, adolescents and young adults up to the age of 25 years, and of the challenges associated with these interventions.

### 4.4 Recommendations for future research

Although progress has been made in understanding the effects that can be achieved from patient education intervention for children, adolescents and young adults, much is yet to be learned. We need more knowledge on the effect of participation over time, how the need for knowledge and education changes at different phases of psychosocial development and illness trajectories, and how patient education interventions can best be tailored to children, adolescents and families with different learning styles and cultural backgrounds. To date, most of the research studies are from interventions for participants with asthma or diabetes. Future studies are recommended on interventions across diagnoses and from a wider range of diagnoses. The authors in several of the included studies highlight that the samples represent a relatively narrow range of socioeconomic status and cultural backgrounds. Thus, future research can benefit from exploring the sustained impact of patient education interventions for children, adolescents and young adults living in a different cultural, ethnic or socioeconomic environment. They should also include psychosocial adjustment and family functioning as intermediate variables.

This review highlights the need for a comprehensive approach in evaluating patient education interventions tailored to children, adolescents and young adults. As is evident from Table 4, the ways of measuring outcomes differ greatly. We need more knowledge about how we can evaluate impact, both for outcomes with standardized measurements, and on how we can evaluate process and subjective experiences from participating in patient education interventions. More consistent use of standardized measurements would also facilitate comparing interventions internationally.

There is a paucity of research on psychological and emotional experiences of children, adolescents and young adults becoming more actively involved in improving their own health. Based on the results from this review, we need more insight into the psychosocial and subjective experience for children, adolescents and young adults, and more knowledge about factors that may sustain or hinder engagement.

### 4.5 Implications for practice

The findings from the present scoping review give important input to political decision makers and health administrators. Most importantly, patient education interventions targeting children, adolescents and young adults can reduce the cost of care and improve the levels of physical activity, BMI and blood glucose control. Moreover, the participants experience beneficial effects owing to less symptom distress and improved knowledge.

This review supports the utility of patient education interventions that employ behavioural strategies tailored to the developmental needs of children, adolescents and young adults with different cultural backgrounds. Such interventions should be made available to a broader range of children, adolescents and young adults who are living with health challenges.

## CONFLICT OF INTEREST

There are no financial or other ties involved in the present work that might lead to a conflict of interest.
