# Supplementary material for: How can we support children, adolescents and young adults in managing chronic health challenges? A scoping review on the effects of patient education interventions
Source: Health Expect. 2019 May 26;22(5):849–62. doi: 10.1111/hex.12906 (PMC6803408; doi:10.1111/hex.12906)
Supplement: Supplementary file 1 [file HEX-22-849-s001.docx]

| First Author, Year, Country | Type of Chronic Illness or Impair-ment Loss | Study Design and Time-Span  | Patient Education Intervention Program | Description of Outcomes | Author Conclusion |
| --- | --- | --- | --- | --- | --- |
|  |  |  |  |  |  |
| Al-sheyab, Gallagher, Crisp, & Shah, 2012  Australia, Jordan | Asthma | RCT (3 months), control group: ordinary care | *Intervention:* Adolescent Asthma Action program. Peer-led education  *Description:* theory-based intervention covering such topics as asthma, its triggers and management, barriers to optimal management including risk-taking behaviors such as smoking  *Mode:* group  *Personnel:* Bilingual health workers, peer leaders, members of the school community  *Delivery method:* face to face, lessons, group discussions, videos, games, and problem-solving sessions  *Duration:* three 45 minutes sessions (during 6 months)  *Age:* 8-10 years  *Setting:* school | - Quality of life - Asthma knowledge | - The intervention gave important improvements on all outcomes measured: health-related quality of life, self-efficacy to resist smoking, and knowledge of self-management of asthma - The outcomes confirm that the program can be modified for Arabic-speaking cultures without loss of effectiveness |
| Almomani et al., 2017  Jordan | Asthma | Prospective RCT (6 months), control group: ordinary care | *Intervention:* clinical pharmacist’s intervention  *Description:* education and pharmaceutical care involving asthma symptoms and triggers, proper use of asthma inhaler devices, asthma medications and how they work, the importance of adherence to asthma medications and, self-management.  *Mode:* individual, patients and caregivers  *Personnel:* pulmonologists, clinical pharmacist  *Delivery method*: face to face, verbal and written information, photos  *Duration:* 6 months  Age: 7-18 years  *Setting:* hospital, outpatient and inpatient | - Asthma control - Quality of life - School absence - Hospital admissions - Emergency department visits - Medication adherence | - Implementation of clinical pharmacy service can positively influence asthma control, child’s and caregiver’s quality of life, and other clinical parameters; it decreased the number of asthma exacerbations, the need for rescue medications, and reported medication side effects and promoted better adherence to medications - Fewer visits to Emergency Department, less rescue steroid usage and school absence |
| Altundag & Bayat, 2016  Turkey | Diabetes 1 | Longitudinal (9 months), control group: ordinary care | *Intervention:* Peer interaction and group education  *Description:* Education focusing on training, social adaptation, peer interaction and practice to gain experience in social interaction  *Mode:* group  *Personnel:* dietitian, diabetes nurse, child psychiatrist  *Delivery method*: face to face and education guide, methods included games, narrating, question-answer, demonstration, discussion, role-play  *Duration*: 4 training sessions of 35-45 minutes, 6 months for social adaptation and social interaction  *Age:* 12-14 years  *Setting:* hospital | - Social support - HbA1c levels - Knowledge levels - Self-esteem | - Decrease in HbA1c levels and an increase in self-esteem and mean scores of social support, and significant increase in knowledge levels and self-respect scores after 9 months |
| Arikan-Ayyildiz et al., 2016  Turkey | Asthma | RCT (3 months), control group: ordinary care | *Intervention:* asthma education program  *Description:* based on the goals of the education program stated by guidelines. Included information on the pathogenesis of asthma, environmental triggers for asthma exacerbations, medications and inhaling technique.  *Mode:* group with parents  *Personnel:* trained educator  *Delivery method:* face to face, videos and visual presentations, individual questions, discussions  *Duration:* 1 hour  *Age:* 5-18 years  *Setting:* hospital | - Asthma control - Exacerbations of asthma symptoms - Hospital admissions - Emergency department visits - Unscheduled hospitalizations - Missed school days | - No significant change of asthma control between education and usual care groups - Health care utilizations and self-reported exacerbations in the study groups were similar, whereas the usual care group reported significantly more missed school days |
| Bean et al., 2015  USA | Obesity | RCT (6 months), control group: ordinary care | *Intervention:* MI Values: Motivational interviewing  *Description:* MI implemented as an adjunct to multidisciplinary treatment. Includes physical activity, dietary intervention and behavioral support  *Mode:* individual, with and without parents  *Personnel:* interventionists trained in motivational interviewing, dietitian and behavioral support specialist  Delivery method:  *Duration:* 2 x 30 minutes, 10 weeks  *Age:* 11-18, mean 13.8 years  *Setting:* tertiary care | - BMI - Adherence to sessions (gym, dietitian and behavioral support) | - Compared with controls, intervention participants had greater 3-month adherence overall (89.2% vs. 81.0%, P = 0.040), to dietitian support (91.3% vs. 84.0%; P = 0.046) and behavioral support visits (92.9% vs. 85.2%; P = 0.041). Their 6-month adherence was better overall (84.4% vs. 76.2%, P = 0.026) as was their adherence to behavioral support visits (87.5% vs. 78.8%, P = 0.011) |
| Bowen, 2013  USA | Asthma | RCT (6 weeks), control group: ordinary care | *Intervention:* modified Open Airways for Schools (OAS)  *Description:* theory-based asthma education program about asthma, sharing feelings and a deep-breathing exercise. Topics included problem solving, review of medications, how to respond during exacerbations, and ways to stay active  *Mode:* group  *Personnel:* intervention trainer  *Delivery method:* face to face, discussions, exercises, attendance card (this was intended to increase the child’s sense of involvement and provide positive reinforcement for coming to the sessions), healthy snacks, completion certificate, gym bag  *Duration:* three weekly sessions of 90 minutes  *Age:* 8-12 years  *Setting:* school | - Asthma control - Forced expiratory volume in 1 second - Asthma knowledge - Quality of life | - The intervention group demonstrated significant and sustained improvement in asthma knowledge - There was no significant difference between groups for quality of life or pulmonary function - While the intervention is a good program for asthma education, the program alone may not be sufficient to combat risk factors associated with poor pulmonary function |
| Bryant-Stephens & Li, 2008  USA | Asthma | RCT (12 months): control groups: ordinary care (historical controls and matched controls for each subject) | *Intervention:* Home-Based Environmental Remediation  *Description:* 2 interventions: home visits only or home visits with environmental remediation. Asthma education covering pathology, signs, symptoms, triggers, review of medication, avoidance techniques, medicine action plan  *Mode:* individual with the caregiver  *Personnel:* lay educators  *Delivery method*: face to face education, home assessment and instructions, equipment and demonstrations  *Duration:* 2 months, 5 visits  *Age:* 2-16, mean age 6 years  *Setting:* home | - Length of hospital stay - Emergency department visits - Number of sick visits (related to asthma) - Symptom frequency - Medication management - Trigger reduction | - Both intervention groups experienced reduction of hospitalizations, emergency room visits, sick visits and asthma symptoms - Both groups showed outcomes significantly superior to the matched control group - The intervention effectively reduced the presence of rodents and carpet in home and increased the use of mattress and pillow covers |
| Burkhart, Rayens, Oakley, Abshire, & Zhang, 2007  USA | Asthma | RCT (4 months), control group: ordinary care | *Intervention:* Self-Management  *Description*: asthma education plus contingency management, based on cognitive social learning theory, including self-monitoring, a contingency contract, tailoring, cueing, and reinforcement specific triggers and medications.  *Mode:* individual, with parent, child-friendly intervention room  Personnel: research associate, a registered nurse and asthma educator  *Delivery method:* face to face, Asthma Diary, short educational videos, individualized teaching with behavioral rehearsal, which is teaching, observing, and then practicing, homework, activity book, parent monitoring  *Duration:* five sessions of 60 minutes (during 16 weeks)  *Age:* 7-11, 9.1 years  *Setting:* pediatric practices | - Frequency and adherence to medication use - Wheezing episodes - Asthma episodes | - Children in the intervention group had higher adherence to PEF monitoring after implementation than did children in the control group - Children in both groups benefited from participating in the study (both received information on how to use the peak expiratory flow rate meters and both were encouraged to do so) - The effect of study participation was stronger over time (after week 16) for those who were randomized to the intervention group, as indicated by greater level of adherence |
| Butz et al., 2014  USA | Asthma | RCT (12 months), control group: attention control group | *Intervention:* clinician and caregiver feedback  *Description:* based on the chronic care model. Nurse home visits with caregiver feedback and preventive asthma care visit with nurse and personal care provider  *Mode:* individual with parent  *Personnel*: specially trained community health nurse  *Delivery method*: face to face  *Duration:* three visits  *Age:* 3-10 years  *Setting:* home | - Preventive care provider visits - Emergency department visits | - The intervention did not improve participants’ asthma preventive care compared to that of an attention control group |
| Cabrera et al., 2013  USA | Diabetes 1 | Longitudinal (5 years), control group: non-academic medical center (different setting) | *Intervention:* initial diabetes education  *Description:* intensive educational series, within the requirements of the American Diabetes Association recognition construct, and tailored to the perceived comfort and educational level of each family  *Mode:* individual, family  *Personnel:* pediatric endocrinologists, dieticians, nurse practitioners, dedicated diabetes social worker  *Delivery method:* face to face  *Duration:* 3 days  *Age:* 6.8 years  *Setting:* academic medical center | - Glycemic control | - No significant differences in glycemic control were found with different setting for the intervention for patients overall, or at 2, 3, or 5 years from diagnosis, even when adjusted for the “time” effect |
| Campbell et al., 2015  USA | Asthma | RCT (12 months), control group: ordinary care | *Intervention:* Healthy Homes program  *Description:* streamlined version of an evidence-based community health worker asthma home visit program. The intervention was tailored to the topics most relevant to each participant using motivational interviewing methods.  *Mode:* individual  *Personnel:* community health worker  *Delivery method:* face to face, telephone calls, e-mail  *Duration:* four home visits  *Age:* 3-17 years  *Setting:* home | - Asthma symptoms - Urgent health services use (asthma-related) - Medication use - Asthma control - Activity | - The intervention improved health outcomes and yielded a monetary return on health care investment of 1.90 - For improved quality of life, the difference across study groups was 0.9 points greater (P = .034) if the child’s caretaker had less formal education; for symptom-free days it was 3.7 days greater (P = .039). No other interactions were significant - Most outcomes improved significantly more in the intervention group than in the control group |
| Cardoso Tde et al., 2014  Brazil | Bipolar disorder | RCT (6 months), control group: ordinary care | *Intervention:* Psychoeducation  *Description:* Combined intervention (psychoeducation plus medication). Theory-based, focusing on nature of the disorder, relieving guilt and clarifying questions regarding the disorder, how an episode occurs, identify the symptoms of these episodes and to learn how to detect its beginning, indication of symptoms and relapses. Leading to structured action plan. Learning of techniques and resources, and importance of adhering to pharmacological treatment.  *Mode:* group  *Personnel:* graduate research students in the last year of Psychology.  *Delivery method:* face to face  *Duration:* six sessions of 1 hour  *Age:* 18-29, mean age 24 years  *Setting:* hospital | - Health related quality of life - Severity of depressive symptoms - Severity of manic and hypomanic symptoms | - There was a significant improvement related to quality of life, both in individuals who received psychoeducation and those who were assigned to treatment-as-usual - The intervention was not more effective than treatment-as-usual in improving perceived quality of life in young individuals |
| Chalder, Deary, Husain, & Walwyn, 2010  UK | Chronic fatigue syndrome | RCT (12 months), control group: psycho-education | *Intervention:* family-focused cognitive behavior therapy  *Description:* theory-based treatment with specific concerns of the parents and sibling. Aims at encouraging the participant to achieve balance between activity and rest, gradually increasing activities including home, social and school life, establishing a sleep routine, addressing fears and negative thoughts, encouraging individuals within the family to express their own views about the illness and agreeing a way forward and paying attention to relapse prevention. supporting parents during transition periods  *Mode:*  *Personnel:* Two trained and experienced cognitive behavioral psychotherapists  *Delivery method*: face to face, homework, treatment guide  *Duration:* thirteen sessions of 1 hour (during 6 months)  *Age:* 11-18 years  *Setting:* | - Attendance at school, college or work - Fatigue - Health related quality of life - Work and social adjustment - Strengths and difficulties - Global satisfaction | - The intervention was no more effective than psychoeducation in improving school attendance, fatigue and social adjustment at the 6-month follow-up - There was a non-significant trend for the intervention group to report better satisfaction than the psycho-education group at the 6-month follow-up, but not after 12 months |
| Chiang, Ma, Huang, Tseng, & Hsueh, 2009  Taiwan | Asthma | RCT (3 months), control group: self-management only | *Intervention*: asthma self-management program  *Description*: combined self-management and relaxation-breathing training. Education about reforming asthma cognition, correct use of asthma drugs, establishing a safe home environment, monitoring with a peak flow meter, and keeping an asthma diary.  *Mode:* individual with parent  *Personnel:* nurse scientist and nursing graduate student  *Delivery method:* face to face and CD, educational booklet, peak flow meter records  *Duration:* 30 minutes, three times a week, 12 weeks  *Age:* 6-14 years  *Setting:* hospital and home | - Anxiety - Asthma symptoms - Self-perceived health status - Peak expiratory flow rate - Asthma medication use | The results indicate that relaxation-breathing training combined with a self-management program can improve both physiological indicators (self-perceived health status, asthma signs/symptoms, peak expiratory flow rate, and reliance on asthma medications) and psychological indicators (anxiety) among children with moderate-to-severe asthma |
| Clark et al., 2010  USA | Asthma | RCT (24 months), control group: self-management with a peer component | *Intervention:* Open Airways for Preteens  *Description:* Self-Management With and without a Peer Component. Self-management: interactive problem-solving activities appropriate for groups of preteens. The topics in the lessons included basic information and feelings, medicines and their use, monitoring one’s physical activity, managing an asthma attack at home or school, deciding when to go to the doctor and making the doctor visit more effective, how to keep yourself healthy, including smoking avoidance, personal characteristics, actions, and environmental factors that lead to successful asthma control. Peer Component focused on enabling students to understand and support their classmates with asthma  *Mode:* group  *Personnel:* graduate students and community leaders with program training, health education coordinator, school personnel. 18% of peer leaders had asthma.  *Delivery method:* face to face, Take-home assignments and handout materials for parents  *Duration:* eleven sessions of 2 hours (during 6 weeks)  *Age:* mean age 11.6 years  *Setting:* schools | - Asthma symptoms - Asthma-related quality of life - Self-regulation - Academic grades | - Neither intervention produced significant change in asthma symptoms or quality of life - One improved school grades (p = 0.02). The other enhanced self-regulation (p = 0.01) at 24 months - Both slowed the decline in self-regulation in undiagnosed preteens at 12 months and increased self-regulation at 24 months   (p = 0.04; p = 0.003) |
| Coughlin et al., 2017  USA | Organ transplant | Longitudinal (12 months), control group: baseline only  (children in the dermatology clinic without solid organ transplant) | *Intervention:* multimodal educational intervention  *Description:* Based on guidelines. Information about skin cancer risk, recognizing skin cancer, effective sun-protection behaviors, and skin self-examination  *Mode:* individual with guardians  *Personnel:* dermatologist (from supplementary material)  *Delivery method*: face to face with verbal instruction, questions and discussions, a video, and printed materials to take home  *Duration:* 1 session of 15-20 minutes  *Age:* 10-17, mean age 13 years  *Setting:* hospital | - Sun-protection behavior - Self-efficacy | - The participants demonstrated significantly increased knowledge 1 week after their educational session, and gains persisted to the 6-month follow-up - Although the participants’ perceived barriers to sun-protection behaviors did not change significantly over follow-up, they reported significant improvements in sun-protection behaviors |
| Curtis et al., 2016  Australia | First episode psychosis | Longitudinal study (4 months), control group: ordinary care | *Intervention:* keeping the body in mind  *Description:* holistic, individualized lifestyle and life skills intervention based on guidelines for the prevention of weight gain. Health coaching with goal identification and structured motivational interviewing, and supervised exercise prescription  *Mode:* individual  *Personnel:* specialist clinical staff (nurse, dietician and exercise physiologist) and youth peer wellness coaches  *Delivery method:* face to face  *Duration*: 12 weeks  *Age:* mean age 20 years  *Setting:* community center | - Weight - Blood pressure - Self-esteem - Sleep-quality - Function - Medication adherence - Physical activity - Nutrition | - A lifestyle and life skills intervention delivered as part of standard care attenuated antipsychotic-induced weight gain in young people with first episode psychosis - The intervention was acceptable to the young people referred to the service - Such interventions may prevent the seeding of future disease risk and in the long term help reduce the life expectancy gap for people living with serious mental illness |
| Davis, Benson, Cooney, Spruell, & Orelian, 2011  USA | Asthma | Longitudinal (12 months), control group: ordinary care | *Intervention:* bedside education  *Description:* covered the basic physiology of asthma, the role of medications and the asthma action plan, warning signs, communicating with the provider, identifying, avoiding, and remedying asthma triggers, and brief interactive training on delivery devices. Topics such as tobacco cessation and avoidance, nebulizers, and insurance were addressed.  *Mode*: individual, family  *Personnel:* trained respiratory professional  *Delivery method*: face to face with didactic session using photos, demonstration devices and lung models, phone calls, booklet  *Duration:* 45 minutes  *Age:* 1-18 years  *Setting:* hospital | - Hospital days - Emergency department visits | - No significant improvement was observed in the intervention participants or any subgroups followed for 12 months after the intervention |
| Dingemann et al., 2017  Germany | Esop-hageal atresia | Longitudinal (1 month), control group: ordinary care | *Intervention:* ModuS-T: transition-specific educational program  *Description*: generic apart from the condition-specific component “Doc special”: parental training component supported by an interactive web site  *Mode:* group with parents  *Personnel:* psychologist, pediatric surgeon conducted the condition-specific modules (train the trainer)  *Delivery method:* face to face and web site  *Duration:* twelve sessions of 45 minutes during two days  *Age:* mean age 18.3 years  *Setting*: hospital | - Patient activation - Health-related quality of life - Transition specific knowledge - Subjective satisfaction | - Overall, 90% of the patients anticipated a positive effect on the future course of the disease - Patient’s transition-specific knowledge was low before the program. It improved by 18% after the intervention. It did not change in the control group - Patient activation and quality of life were not affected by the intervention |
| Espinoza-Palma et al., 2009  Chile | Asthma | RCT (12 months), control group: ordinary care | *Intervention:* Asthma Education with and Without a Self-Management Plan  *Description:* Information aiming at teaching the participants and their families how to recognize an asthma exacerbation and what to do in different scenarios  *Mode:* Individual conversations with family  *Personnel:* research nurse  *Delivery method:* Face to face, booklet, guide, puzzle game  *Duration:* 30 minutes (face to face component)  *Age:* 5-15, mean age 8 years  *Setting:* hospital | - Hospitalization - Emergency department visits - Asthma exacerbations - Therapy use | - Asthma education with or without a self-management plan during asthma hospitalization was effective in reducing exacerbations, emergency visits, oral steroid burst uses, and future rehospitalizations - The evidence supports the importance of providing a complete asthma education plan for any patient who is admitted for asthma exacerbation |
| Fincher, Shaw, & Ramelet, 2012  Australia | Preo-perative, across diagnosis | RCT (2 weeks), control group: ordinary care | *Intervention*: preoperative preparation  *Description*: structured preoperative preparation  *Mode:* individual, with carers  *Personnel:* trained hospital play specialists  *Delivery method*: face to face with photo file, demonstration of equipment with role-modelling, tour and education kit  *Duration:* 60 minutes  *Age:* 3-12, mean age 6.7 years  *Setting:* hospital | - Child temperament - Anxiety - Pain | - Preoperative preparation was more efficient on parent than child - Although the preoperative preparation had limited effect on child anxiety, it permitted decreased pain experience in the postoperative period |
| Flapper, Duiver-man, Gerritsen, Postema, & van der Schans, 2008  Nether-land | Asthma | RCT (9 months), control group: ordinary care | *Intervention:* education-exercise program  *Description*: theory-based training program with group education/exercise for the child, and education to caregivers and schoolteachers. Designed specifically for the school age group. Exercise program had no focus on improving exercise tolerance, but individual advice and reassurance on capability to perform activities in daily life and sports were offered  *Mode:* group, child with parent and teacher  *Personnel*: pediatric nurse and child physiotherapist trained in the original educational program  Delivery method*: face to face with two*-way dialogue, games and learning materials  *Duration:* 3 months: Children: ten weekly sessions of 2.5 hour (1.5 hour education, 1 hour exercise), parents: five 1.5 hour sessions, teachers: one 1.5 hour  *Age:* 8-12, mean age 10 years  *Setting:* pediatric care, tertiary ambulatory clinic | - Health-related quality of life - Lung function - Sick days - Oral prednisone courses - General practice visits | - Changes in health-related quality of life were clinically important and significantly greater in the intervention group than in the control group - Changes in sick days, oral prednisone courses and doctor visits over a 6-month period were greater in the intervention group than in the control group - Changes could not be ascribed to change in lung function or medication |
| Garanty-Bogacka et al., 2011  Poland | Obesity | Longitudinal (6 months), control group: none | *Intervention:* lifestyle modification intervention program  *Description:* combined hypocaloric diet (reduces fat and sugar) and moderate physical activity (physical exercise as part of everyday life, reducing amount of time spent watching television or playing computer games). Nutrition education, behavioral therapy including individual psychological care  *Mode:* individual, with and without parent  *Personnel*:  *Delivery method*: face to face  *Duration*: 6 months  *Age*: 8-18 years  *Setting*: outpatient clinic | - Blood inflammatory markers - Weight | - This study demonstrates that weight reduction after successful lifestyle intervention results in improvements of blood inflammatory markers in obese children and adolescents |
| Garvik, Idsoe, & Bru, 2014  Norway | Depression | Longitudinal (6 months), control group: none | *Intervention:* Adolescent Coping with Depression Course  *Description:* theory-based course teaching toolbox of skills and techniques in order to cope better with depressive symptoms in the future. Topics such as how sadness and depression arise, relationship between thinking, emotional experiences and feelings, awareness of how to influence one’s own feelings by self-comfort or relaxation, negative automatic cognitions, identification and change of cognition, recognition of negative automatic thoughts, feeling regulation, positive cognitions and social activity  *Mode:* relaxation exercises, homework, self-monitoring, assignments  *Personnel:*  Delivery method: face to face, course pamphlet to participants, parents and schools, presentation for teachers to use in class to present mental health as a theme  *Duration:* 8 sessions  *Age:*  *Setting:* outpatient clinics | - Depression severity | - The results indicate a significant reduction in symptoms of depression among participants - The reduction in symptoms of depression was equal to the reduction found in previous randomized controlled trials of similar courses, and significantly greater than those found among the control groups in the studies constituting the benchmark - Among the adolescents who completed follow-up questionnaires six months later, the reductions were found to be maintained or even increased |
| Gordon et al., 2015  UK | Autism spectrum disorder | RCT (4 months), control group: no intervention | *Intervention:* psychoeducation group for autism spectrum understanding and support  *Description:* provide information with focus on strengths, encouraging young people to focus on their capacities as well as difficulties. Empower parents to support their child’s learning, to encourage discussion of child strengths, help to cope with the impact of the diagnosis on the family system. Predictably structured and designed to be comfortable, fun and accessible.  *Mode:* group, separate with parents  *Personnel:* clinical psychologists  *Delivery method:* face to face with information, discussions, home tasks, educational games, visual aids and worksheets  *Duration:* 1.5 hours, 6 weekly sessions  *Age:* 9-14, mean age 11.5 years  *Setting:* hospital | - Strengths and difficulties - Self-esteem - Autism knowledge | - After the intervention, participants had more general knowledge about autism spectrum disorder, and showed a greater awareness of their collection of unique strengths and difficulties associated with the diagnosis - Self-reports did not show any effect of the intervention on self-esteem, |
| Grey et al., 2009  USA | Diabetes 1 | RCT (12 months), control group: general diabetes education | *Intervention:* coping skills training (CST) intervention  *Description:* theory-based intervention to increase competence and mastery by retraining non-constructive coping styles and behaviors into more constructive behaviors. Covering introduction, communication, social problem solving, conflict resolution, stress management and self-talk  *Mode:* group, family, children and parents met simultaneously but separately  *Personnel:* CST trainers? clinicians at the recruitment site  *Delivery method*: face to face, role-play, relaxation exercises, discussions, practice games  Duration: six 1.5-hour sessions during six weeks  *Age:* 8-12 years  *Setting:* pediatric diabetes clinic | - Coping - Self-efficacy - Family behavior | - The intervention did not have the expected effect on child and family outcomes in this relatively well-adjusted sample of school-aged children with diabetes - Both the intervention and control group with general diabetes education improved psychosocial outcomes for children |
| Guner & Cele-bioglu, 2015  Turkey | Asthma | Longitudinal (2 months), control group: ordinary care | Interven*tion:* Nursing intervention  *Description:* asthma maintenance, asthma control, training and medication-use implementation  *Mode:* home visits, family  Personnel: research nurse  *Delivery method*: face to face training, VCD, booklet  *Duration*: two sessions of 45-50 minutes during two days, four health center visits  *Age:* 10-18 years  *Setting:* health centers | - Disease evaluation form (asthma symptoms and frequency, school absenteeism, medication usage and emergency service use) - Self-efficacy - Peak Expiratory Flow Rate | - A significant increase in mean self-efficacy score in the intervention group - Reduction in asthma symptoms, less limitations to daily function and fewer attacks following physical activity in the intervention group - Children in the intervention group were more conscious of the symptoms of asthma attacks and used preventive and rescue medications regularly - Fewer absences from school and fewer emergency room visits in the intervention group |
| Haeberli et al., 2008  Switzer-land | Cancer | Longitudinal (pre- and post intervention with two groups) | *Intervention:* psychoeducation  *Description:* tailored psychoeducational intervention to get familiar with staff, equipment and procedure encountered during radiotherapy  *Mode:* individual  *Personnel:* trained nurse  *Delivery method:* face to face with interactive support, play program, reward system, visitations  *Duration:* five sessions of 1.5 hours  *Age:* mean age 9 years  *Setting:* hospital | - Need for anesthesia | - The intervention was able to reduce the need for anesthesia in children undergoing radiotherapy for cancer - This results in lower costs and increased cooperation during radiotherapy |
| Hashemi-pour, Kelishadi, Tavalaee Zavvareh, & Ghatreh-Samani, 2012  Iran | Obesity | Longitudinal (12 months), control group: none | *Intervention*: family-oriented weight reduction program  *Description:* educational program. Dietary recommendation and increasing physical activity  *Mode:* individual, family  Personnel: physician  *Delivery method:* face to face  *Duration:* four sessions of 45-60 minutes  *Age:* 4-18, mean age 9 years  *Setting:* outpatient | - Body mass index - Waist circumference - Hip circumference | - Children’s body mass index z-score decreased significantly after the study - Children waist circumference and hip circumference significantly increased |
| Herbert, Sweenie, Kelly, Holmes, & Streisand, 2014  USA | Diabetes 1 | Qualitative interviews | *Intervention:* family behavioral intervention  *Description:* TeamWork sessions related to daily life of each family. Including topics such as communication, diabetes management, problem solving to improve blood glucose management, healthy food choices and avoiding arguments, how attitudes affect behaviors and how these relate to physical activity  *Mode:* group, family  *Personnel:* study team counselor (nurse scientist or clinical psychologist)  *Delivery method*: face to face with discussions, materials such as study magnets and handouts  *Duration*: 4 sessions  *Age:* 11-14 years  *Setting:* hospital | - Qualitative evaluation | - Five themes: TeamWork content, TeamWork structure, transition of responsibility, current and future challenges, and future intervention considerations - Addressing diabetes challenges as a parent-adolescent dyad via a behavioral clinic program is helpful to families during adolescence |
| Holmes, Chen, Mackey, Grey, & Streisand, 2014  USA | Diabetes 1 | RCT (3 years), control group: education group | *Intervention*: individualized coping skills program (i) and diabetes education treatment (ii)  *Descriptio*n: (i) individualized intensive family teamwork coping skills program with discussions and practice of coping skills, attitudes and behavioral change. Glucose management, problem solving dietary issues, cognitive reframing to promote exercise, parental support and communication. Activities, development of behavioral practice plan, development of individual practice plan for home use. Contact between sessions. (ii) uniform education content including communication about diabetes, diabetes and extracurricular activities, travel, and school issues including education plans and diabetes rights. Brochure of the major session points. No discussion of parental involvement, authoritative parenting, or practice plans. No contact between sessions  *Mode:* individual, family: one parent and one youth. Before or after medical appointment  *Personnel:* (i) graduate-level interventionists with three days training. (ii) bachelor-level facilitators with knowledge of pediatric diabetes and experience with families, received supervised training throughout course  *Delivery method:* face to face and written materials, (and telephone follow-up in coping skills intervention (i), not in diabetes education intervention (ii))  *Duration:* (i) four sessions of 30-45 min, (ii) four sessions of 15-20 min during one year  *Age:* 11-14 years  *Setting:* hospital | - Socioeconomic status - Glycemic control - Diabetes adherence - Family conflict - Self-efficacy | - Both treatment strategies successfully prevented deterioration in adolescent disease care and simultaneously improved adolescent quality of life - The education strategy was more efficacious than the coping strategy in improving diabetes adherence and glycemic control over a 3-year follow-up |
| Horner & Fouladi, 2008  USA | Asthma | RCT (6 weeks), control group: attention control | *Intervention:* Asthma self-management  *Description:* lay health educator-delivered classes about an asthma self-management plan designed for rural children. Content included information on asthma pathophysiology, symptoms, and management; skills practice to increase self-efficacy of asthma management  *Mode*: group  Personnel*: lay health educators* nominated by school personnel, trained in separate sessions by the principal investigator to deliver their respective curricula.  *Delivery method:* face to face, MDI and peak flow meters and vignettes  *Duration:* sixteen sessions of 15 minutes, twice or thrice a week  *Age:* mean age 8.7 years  *Setting:* schools | - Knowledge - Asthma self-management - Asthma self-efficacy - Metered dose inhaler technique | - The delivery of an asthma health education intervention by trained lay health educators to school-aged children was an effective means to improve children’s asthma knowledge, asthma self-management, self-efficacy for managing asthma symptoms, and metered dose inhaler technique. There were significant group interaction effects for the treatment intervention on the measures of children’s asthma knowledge, asthma self-management, and metered dose inhaler technique |
| Indinni-meo et al., 2009  Italy | Asthma | RCT (12 months), control group: ordinary care | *Intervention:* Long term education program  *Description:* developed by a scientific board, covers basic information on asthma and on preventive measures to identify and control trigger factors, learning to recognize the first symptoms of asthma and treat acute asthma, how to use asthma medications and healthcare facilities, and strategies for remaining active, avoiding asthma triggers, complying with therapy and engaging in outdoor activities  *Mode:* group, separate and jointly with parents  *Personnel*: resident physicians and nurses who received training beforehand  *Delivery method:* education and discussions, jigsaw puzzles, asthma-themed playing cards, coloring book and match-pairs game  *Duration:* one hour separate, thirty minutes joint interaction  *Age:* mean age 8.8 years  *Setting:* clinics | - Asthma attacks - Use of medication - Unscheduled visits to family physician - Emergency Department visits | - Participants in the intervention group reported significantly fewer asthma attacks after 12 months than controls - No significant difference found in Emergency Department visits and school absences - For the subgroup of children who had 3 or more asthma attacks at baseline, parents’ knowledge improved significantly more in the educational group than in the control group |
| Janssens & Harver, 2015  Belgium | Asthma | Mixed methods (RCT, pre- and post-measures with three groups and interviews after RCT) | *Intervention:* Providing feedback for patient estimates of peak expiratory flow rate (PEFR)  *Description:* pediatric asthma research program of asthma education, home monitoring, resistive load detection sessions, and six-month follow-up. PEFR training with feedback, PEFR training without feedback, and no PEFR. All conditions involved asthma symptom diary  *Mode:* individual  *Personnel:* health care providers (?)  *Delivery method*: face to face, monitoring  *Duration:* three sessions of asthma education, two cycles of home monitoring of asthma symptoms for thirty days, four resistive load detection sessions, and six-months follow-up  *Age:* 8-15, mean age 10 years  *Setting:* home | - Quality of life - Asthma trigger identification - Experiences during the home monitoring period | - The intervention resulted in increased reported triggers, which increased reliably as a function of home monitoring, and increased further in participants who completed discrimination training with feedback - Increases in the number of reported asthma triggers were associated with decreases in quality of life |
| Julian et al., 2014  France | Asthma | Longitudinal (3 months), control group: none | *Intervention:* therapeutic education (TE) program  *Description:* general explanation of the disease and how it is treated, Written Action Plan (WAP) (i) individual educational diagnosis (ii) pathophysiology of asthma, the symptoms of the disease, the triggering factors, the prodromal and clinical signs of an attack and the treatments (iii)  *Mode:* individual with parent and group; individual with parent (i), individual with parent (ii), group, families (iii)  *Personnel:* pediatric pulmonologist, doctor with therapeutic education program experience,  *Delivery method:* face to face  *Duration:* 3.5 hours; 30 minutes (i), 30 minutes (ii), 2.5 hours (iii)  *Age:* mean age 8.1 years  *Setting:* hospital | - Quality of life - Asthma management - School/ workplace absenteeism - Functional respiratory parameters | - The intervention did not significantly alter the children’s quality of life score in the different areas studied: autonomy, leisure activities, functions, and social relations - These results were accompanied by a significant improvement in asthma management, with, in particular, a major decrease in the use of medication, and the number of unscheduled medical consultations and visits to the emergency department; a decrease in school absenteeism; and an improvement in forced expiratory volume in 1 sec |
| Katz, Volken-ing, Butler, Anderson, & Laffel, 2014  USA | Diabetes 1 | RCT (2 years), two groups compared with ordinary care group | *Intervention*: Care ambassador and family-based psychoeducation  *Description:* pediatric diabetes subspecialty care including basic care coordination by the care ambassador (ordinary care) (i) + monthly outreach by the care ambassador via phone or email (ii) + psychoeducational intervention with problem-solving exercises and role-playing of realistic expectations for family teamwork (iii)  *Mode:* individual, family  *Personnel:* care ambassador (research assistant with a 4-yr college degree and no medical background who was trained in study protocol implementation and care coordination  *Delivery method:* face to face, phone, e-mail  *Duration:* 30-min sessions quarterly (iii)  *Age:* 8-16 years  *Setting:* hospital | - Parental involvement - Family conflict - Quality of life - Body mass index - Blood glucose monitoring | - Blood glucose monitoring showed no differences across treatment groups - Among youth with suboptimal blood glucose levels at baseline, more in the psychoeducation group (iii) maintained or improved their blood glucose monitoring, and maintained or increased parent involvement than in the other two groups combined (i+ii), without negative impact on youth quality of life or increased diabetes-specific family conflict |
| Kelsey et al., 2016  USA | Diabetes 2 | Longitudinal (2 months), control group: none | *Intervention:* short-term standardized diabetes education and treatment with metformin (Today)  *Description:* individualized standardized diabetes education including diabetes pathophysiology, medication action, lifestyle guidelines, diabetes self-care and goal-setting  *Mode:* individual, with family member  *Personnel*: certified diabetes educator  *Delivery method:* face to face  *Duration:* six to twelve visits during two to six months  *Age*: 10-17 years  *Setting:* hospital | - Biochemical - Anthropometrics | - Treatment with metformin and diabetes education provided short-term improvements in glycemic control and cardiometabolic risk factors in a large adolescent type 2 diabetes cohort - Nearly all insulin-treated youth could be successfully weaned off insulin with continued improvement in glycemic control |
| Kenney et al., 2016  USA | Diabetes (prevention) | Longitudinal (12 months), control group: none | *Intervention:* together on diabetes  *Description:* family-based diabetes prevention and management program. Home-based education and support, collaboration with medical providers, and referrals to community resources and wellness events. Encouraged youth to set small obtainable goals regarding nutrition, physical activity, and life skills while providing the knowledge and skills needed to reach those goals. Encouraged support persons to gain knowledge and skills related to household behavior change  *Mode*: individual with support person  *Personnel*: family health coaches  *Delivery method:* face to face  *Duration:* 6 months intervention: twelve sessions of 45-60 minutes and six follow-up sessions of 20 minutes for youths, four sessions of 20-30 minutes for support persons  *Age*: 10-19 years  *Setting:* home-based | - Quality of life - Family involvement - Physical activity - Diabetes prevention and management - Nutrition - Social skills knowledge - Body mass index - Blood pressure - Blood glucose monitoring | - At 12 months post-enrollment, improvements were observed in youth’s quality of life, depressive symptoms, knowledge related to the intervention content, standardized body mass index scores, and hypertension - Improvements in mean blood glucose levels were observed among diabetic youth with baseline blood glucose >6.5% (P = 0.036) |
| Krieger, Takaro, Song, Beaudet, & Edwards, 2009  USA | Asthma | RCT (12 months), control group: ordinary care | *Intervention:* Self-management support  *Description*: based on social cognitive theory and the transtheoretical stages of the change model. Community health workers reviewed participants’ asthma control, self-management practices, and access to medical care. Follow-up visits, social support and advocacy for clients; educational topics such as medication use, action plans, effective use of the medical system, medical adherence, and trigger reduction (i).  *Mode:* individual  Personnel: clinic-based nurses and in-home community health workers (who shared ethnic backgrounds with participants and had personal or family experience of asthma)  *Delivery method:*  *Duration:* one intake and an average of 4.5 follow-up visits during the course of a year as well as interim telephone communication (i): 1 intake and 3 follow-up clinic visits at 3-month intervals (ii)  *Age:* 3-13 years  *Setting:* community health clinics and home | - Asthma symptom-free days - Quality of life - Asthma attacks - Use of medication - Self-management - Missed school/work days - Self-efficacy - Urgent care visits | - Both groups showed significant increases in caretaker quality of life and number of symptom-free days, and absolute decreases in the proportion of children who used urgent health services in the prior 3 months - Quality of life improved by 0.22 more points in the intervention group. The number of symptom-free days increased by 0.94 days per 2 weeks, or 24.4 days per year, in the intervention group - While use of urgent health services decreased more in the intervention group, the difference between groups was not significant |
| Laguna-Alcaraz, Mejia-Rodri-guez, Rendon-Paredes, Villa-Barajas, & Paniagua, 2017  Mexico | Over-weight and obesity | Longitudinal (6 months), control group: none | *Intervention:* educational sessions for self-care, physical activity and nutritional counseling  *Description*: Lifestyle educational sessions covered such topics as health promotion, nutrition, prevention and control of diseases and sexual and reproductive health. Workouts for physical activity sessions were designed for the whole family, in which the interaction of parents with children was favored.  *Mode*: individual, family  *Personnel:* general undergraduate physician following the educational strategy provided by the program, undergraduate general practitioner, expert in physical education  Delivery method: face to face  *Duration*: lifestyle sessions once a week for one hour, workout sessions three times a week for one hour during six months  *Age:* mean age 11.5 years  *Setting:* primary care setting | - Lifestyle - Body mass index - Waist circumference - Blood pressure - Fasting blood glucose | - The lifestyle was improved after the intervention in the domains of family and friends, nutrition and alcohol intake - Body mass index, waist circumference decreased - Cardiovascular risk factors and metabolic syndrome decreased significantly |
| Larson et al., 2010  Australia | Asthma | Longitudinal (12 months), control group: none | *Intervention*: Nurse-led patient education intervention  *Description:* research-based intervention developed by multidisciplinary team covering pathophysiology of asthma, asthma signs and symptoms, trigger factors, education in the use of medications and importance of preventer adherence, assessment and training in correct device technique, the four step emergency plan, and advice on exercise induced asthma and smoking cessation when relevant, Asthma Action Plan  *Mode*: individual  *Personnel:* nurses accredited asthma educators  *Delivery method:*  *Duration*: one session of 20 minutes  *Age*: 7-82 (25% of the patients were under 16 years of age)  *Setting:* general practice | - Medication use - Asthma control - Quality of life - General practice visits - Asthma action plans - Emergency Department visits | - Mean asthma control score improved but the change did not reach statistical significance - The proportion of patients who had at least one unscheduled visit to their general practitioner over 12 months decreased from 23% to 13% - Emergency department presentations decreased from 9% to 4% |
| Li, Chung, Ho, Chiu, & Lopez, 2013  China | Cancer | RCT (9 months), control group: placebo (same time and activities) | *Intervention:* integrated adventure-based training and health education program  *Description:* research-based intervention with education leading to an individual action plan for regular physical activity tailored to participants who did not engage in regular physical activity  *Mode:* group  *Personnel:* healthcare professionals working in a local university, adventure-based training instructors  *Delivery method:* face to face with workshop, educational talks, adventure-based training activities  *Duration:* 4 days  *Age:* 9 16, mean age 12 years  *Setting:* day camp training center | - Physical activity - Physical activity self-efficacy - Quality of life | - Participants in the intervention group reported statistically significant differences in physical activity stages of change, higher levels of physical activity and self-efficacy than those in the control group - There were statistically significant mean differences in physical activity levels, self-efficacy, and quality of life of participants in the intervention group from baseline to 9 months after starting the intervention |
| Lloyd, Chalder, & Rimes, 2012  UK | Chronic fatigue syndrome | RCT (2 years), control group: psychoeducation | *Intervention:* Cognitive behavioral therapy (CBT) or psychoeducation  *Description*: family-focused CBT to treat chronic fatigue syndrome (CFS) developed with the aim of encouraging a balance between activity and rest, gradually increasing activities, establishing a sleep routine, addressing unhelpful beliefs, encouraging family members to express their own views about the illness and agreeing on a way forward, and paying attention to relapse prevention. (i). The psychoeducation control used similar methods and a checklist, which ensured that therapists gave the message that untreated CFS in adolescents has a good prognosis, presented a model of CFS that distinguished predisposing, precipitating and maintaining factors, introduced the concept of symptom management, i.e. that the way that physical symptoms are managed makes a difference to outcome, physical illness analogies such as heart disease were used to increase likelihood of engagement, advice on pacing and consistency of activity and rest, advice on sleep management, conveying the message that increased symptoms do not mean more pathology and advice on gradually building up activity over a period of months (ii).  *Mode:* individual, family  *Personnel:* trained cognitive behavior psychotherapists.  *Delivery method*: face to face, treatment manual, homework (i): didactic with discussion, information giving and problem solving (ii)  *Duration:* thirteen sessioins of one hour during seven weeks (i), four sessions of one hour during six months (ii)  *Age:* 11-18, mean age 15 years | - School attendance - Fatigue - Physical functioning - Impairment - Strengths and difficulties | - The proportion of participants reporting at least 70% school attendance at 24 months was 90% in the CBT group and 84% in the psycho-education group - The proportion of adolescents who had recovered in the family-focused CBT group was 79% compared with 64% in the psycho-education group, according to a definition including fatigue and school attendance - Family-focused CBT was associated with significantly better emotional and behavioral adjustment at 24 month follow-up compared to psycho-education, as reported by adolescents - Impairment significantly decreased in both groups between the 6- and 24-month follow-ups, with no significant group difference in improvement over this period - Gains previously observed for other secondary outcomes at the 6-month follow-up were maintained at 24-month follow-up with no significant further improvement or group differences in improvement |
| Magza-men, Patel, Davis, Edelstein, & Tager, 2008  USA | Asthma | Longitudinal (3 months), control group: none | *Intervention*: Kickin´asthma  *Description*: asthma curriculum designed by health educators and local students. Teaches asthma physiology and asthma self-management techniques. Structurally similar to “Open Airways for Schools”, covering lung physiology and asthma basics, triggers, symptoms, and warning signs; medication; emergencies, problem solving, and review.  *Mode:* group,skits, games, videos, and role-playing scenarios, reminders, funny invitations  *Personnel:* peer educators and health educators, asthma nurse  *Delivery method*:  *Duration*: four weekly sessions of 50 minutes  *Age:* 6-18 years  *Setting:* schools | - Symptoms - Emergency Department visits - Hospitalization | - Comparison of baseline to follow-up data indicated that students experienced significantly fewer days with activity limitations and significantly fewer nights of sleep disturbance after participation in the intervention - For health care utilization, students reported significantly less frequent emergency department visits or hospitalizations between the baseline and follow-up surveys |
| Majum-dar, Bethin, & Quattrin, 2015  USA | Diabetes 1 | RCT (12 months): control group: ordinary care | *Intervention:* Enhanced Dietary Counseling  *Description*: standard dietary counseling plus nutritional education and food records to new-onset type 1 diabetes  *Mode:* individual  *Personnel*: registered dietician (RD)/ Certified Diabetes Educator (CDE)  *Delivery method*: face to face and telephone  *Duration:* 6 months  *Age:* 8-13 years  *Setting*: hospital outpatient | - Body mass index - Nutrition/daily energy - Blood glucose - Insulin use | - Only a quarter of families recalled the recommended daily energy intake accurately at their 6-week visit despite recent education - The percentage of intervention subjects who recalled the recommended daily energy intake correctly at 6 months after diagnosis improved significantly, unlike in the control group - In contrast, approximately 80% of families in both groups recalled recommended daily carbohydrate intake at 6 weeks |
| Maslow et al., 2013  USA | Chronic illness | Longitudinal, pre- and postsurvey control group: none | *Intervention:* The Adolescent Leadership Council-program  *Description:* hospital-funded program where high school participants and college mentors meet to discuss different aspects of growing up with a chronic illness. Topics covered include diagnosis, living with an illness, interacting with doctors, school issues, friends, and family relationships. Participants act as leaders in designing strategies for reaching out to the broader community to educate other adolescents, parents, or physicians about the topics from discussions  *Mode:* group  *Personnel:* program director. Volunteer staffs include Triple Board, pediatric, and psychiatry residents and other volunteers, such as child life therapists and medical students who help facilitate group discussions. Resident supervision is provided by an attending pediatrician and also by an attending child psychiatrist  *Delivery method:* face to face with dinner groups, open discussion and socialization  *Duration:* 2.5 hours, monthly  *Age:* mean age 15.4 years  *Setting:* hospital | - Loneliness - Competence and confidence - Educational experiences - Medical transition | - The intervention applies the principles of positive youth development to support positive educational, vocational, and health care outcomes for youth with chronic illness - Program development using this perspective is an important new approach for supporting adult development of youth with chronic illness |
| McGhan et al., 2010  Canada | Asthma | RCT (12 months), control group: ordinary care | *Intervention:* Roaring adventures of puff  *Description:* childhood asthma education program. Sessions for children with asthma information, goal setting, use of peak flow meter, diary monitoring, trigger identification, control and avoidance, pathophysiology, medication use, proper medications, symptom recognition, self-monitoring, action plan, lifestyle, exercise, fears, managing asthma episodes, sharing information with teachers and parents. Based on social cognitive theory. Parent and teacher asthma awareness session with needs, environmental control, written asthma action plan for parents. Guidelines at school, communication with physicians.  *Mode:* group  *Personnel*: registered respiratory therapists and community health nurse  *Delivery method*: face to face, puppetry, games, roleplay, model building, group interaction, team building  *Duration*: six sessions of 45-60 minutes  *Age:* mean age 8.6 years  *Setting*: schools | - Quality of life | - The intervention group had more smoke exposure at baseline - Participants lost to follow-up had more asthma symptoms - Improvements were significantly greater in the intervention group from baseline to six months than in the control group in terms of parent’s perceived understanding and ability to cope with and control asthma, and overall quality of life. - On follow-up, doctor visits were reduced in the control group. |
| Melnyk et al., 2015  USA | Overweight and depression | RCT (12 months), control group: attention control program | *Intervention:* COPE healthy lifestyles TEEN program  *Description:* manualized educational and cognitive-behavioral skills building program guided by cognitive theory with physical activity, integrated in health course. Teaches the adolescents that how they think is directly related to how they feel, and how to turn negative beliefs triggered by activating events into positive beliefs so that they feel emotionally better and engage in healthy behaviors. Pedometers serve as cue recognition for increasing physical activity throughout the program and instruct students to increase their step counts by 10% each week regardless of baseline steps. Tracking sheet with averages monitored to physical activity goal.  *Mode:* group  *Personnel:* Teachers with a full-day training workshop on the intervention  *Delivery method:* face to face, homework, manual  *Duration:* 15 sessions  *Age:* mean age 14.7 years  *Setting:* schools | - Body mass index - Depression | - Intervention teens had a significantly lower body mass index at 12 months than control teens - There was a significant decrease in the proportion of overweight and obese teens from baseline to 12 months in the intervention group as compared with control group - For youth who began the study with extremely elevated depressive symptoms, intervention teens had significantly lower depression at 12 months compared with control group |
| Murphy et al., 2012  Australia | Diabetes 1 | RCT (12 months), control group: ordinary care | *Intervention:* family-centered group education program  *Description*: skills training and family teamwork, conventional diabetes self-management education and family communication training  *Mode:* group, with parents  *Personnel:* multidisciplinary health professionals with program-specific training  Delivery method: face to face  Duration: six sessions of 90 minutes, monthly  *Age:* 11-16 years  *Setting*: hospital | - Hypoglycemia - Blood glucose - Quality of life - Health behavior in school children - Diabetes family responsibility questionnaire - Problem areas in diabetes - Diabetes control and complications | - Session attendance was poor, with 48⁄158 families (30.4%) not attending any sessions and only 75⁄158 (47.5%) families attending 4 or more group education sessions - All biomedical and psychosocial outcomes were comparable between groups - At 18 months there was no significant difference in blood glucose in either group and no between-group differences over time - Adolescents perceived no changes in parental input at 12 months |
| Ng et al., 2008  China | Asthma | RCT (11 weeks), control group: wait-list | *Intervention:* We together–We success  *Description*: asthma management with focus on knowledge and skills, and emotion management involving multiple physical, emotional and social dimensions. Discussions on understanding, appreciating child and self, relating, allowing child to grow, hope, self-efficacy.  *Mode:* group with caretaker, separate and joint parts  *Personnel:*  *Delivery method*: face to face with sharing experiences, problem-solving, planning and practicing  *Duration*: 11 weeks, 11 x 2 hours  *Age:* 7-12, mean age 9.2 years  *Setting*: hospital | - Airway inflammation - Forced expiratory volume - Forced vital capacity - Adjustment to asthma | - The results revealed a significant decrease in airway inflammation, and an increase in patient’s adjustment to asthma and parents’ perceived efficacy in asthma management - Serial trend analysis revealed that most psychosocial measures continued to progress steadily after intervention - Significant improvements in both symptom-related measures and mental health and relationship measures were observed |
| Otsuki et al., 2009  USA | Asthma | RCT (18 months), two intervention groups, control group: ordinary care | *Intervention*: asthma basic care and asthma education  *Description:* Asthma basic care and objective feedback on medication adherence with electronic medication monitors, supportive feedback on adherence to encourage a partnership with the family, goal-setting and reinforcement for attaining adherence goals  *Mode:* individual, family  *Personnel:* trained asthma educators  *Delivery method*: behavioral charts and symptom diaries  *Duration*: five sessions of 30-45 minutes  *Age:* mean age 7 years  *Setting:* hospital, home-based | - Medication adherence - Asthma symptoms - Emergency Department visits - Hospitalization - Medication use | - Asthma education led to improved adherence and decreased morbidity compared with usual care - Decreased visits to Emergency Departments - Home-based educational interventions may lead to modest short-term improvements in asthma outcomes among inner-city children - Adherence feedback did not improve outcomes over education alone |
| Powers et al., 2013  USA | Chronic migraine | RCT (12 months), control group: headache education | *Intervention:* Cognitive behavioral therapy plus amitriptyline  *Description*: evidence-based coping skills protocol for pediatric pain, modified to include biofeedback. Behavioral pain management, distraction techniques, relaxation and pleasant activities, activity level and pacing, problems-solving managing headaches and barriers, coping skills, training of cognitive strategies, parents’ role, maintenance plan, parent guidelines.  *Mode*: individual, with and without parent (separate and joint sessions)  *Personnel*: trained study therapists  *Delivery method*: diaries, discussions, home practice, demonstrations  *Duration*: eight sessions of one hour, five booster follow-up sessions  *Age*: 10-17 years  *Setting*: hospital | - Headache symptoms - Treatment integrity - Treatment credibility - Perceived effect of headaches and migraines on school, home, play, and social activities | - Among young persons with chronic migraine, the use of cognitive behavioral therapy plus amitriptyline resulted in greater reductions in days with headache and migraine-related disability compared with use of headache education plus amitriptyline - These findings support the efficacy of cognitive behavioral therapy in the treatment of chronic migraine in children and adolescents |
| Price et al., 2016  UK | Diabetes 1 | RCT (2 years), control group: ordinary care | *Intervention*: Kids in Control of Food  *Description:* structured education course employing interactive and practical learning activities focusing on carbohydrate counting and insulin adjustment in everyday life. The management of hypoglycemia, ketosis and long-term complications of diabetes are considered with scenario-based teaching. Parents took part in one education day before course, covering an overview of carbohydrate counting, insulin dose adjustment, management of hypoglycemia and illness. Parents met the educators at the end of each day, and had an additional session on the final day  *Mode:* group  *Personnel*: research educators (a nurse and a dietician) and one local team member, with 5–day teaching skills course  *Delivery method*: face to face, written material and quizzes  *Duration:* 5 days  *Age:* 11-16 years  *Setting:* hospital, pediatric centers | - Blood glucose - Quality of life - Fear of hypoglycemia - Diabetes self-efficacy - Body mass index | - At 6 and 12 months the intervention group showed significantly improved total generic quality of life scores compared with controls - Across the whole intervention group, mean blood glucose levels were not significantly different from controls |
| Pyatak et al., 2017  USA | Diabetes 1 | RCT (12 months), two intervention groups, control group: ordinary care | *Intervention*: Let’s Empower and Prepare  *Description:* developmentally tailored diabetes education, case management, and clinical care through a program of structured transition from pediatric to adult care  *Mode*: individual  *Personnel*: Case managers with bachelor’s or master’s degrees in public health and previous experience in project coordination for clinical research  *Delivery method:* face to face, reminder calls, carbohydrate counting class, social networking website  *Duration:* quarterly at clinical visits over 12 months  *Age*: 19-25 years  *Setting:* hospital, young adult clinic | - Diabetes care visits - Blood glucose - Episodes of hypoglycemia - Emergency Department visits - Hospitalization - Diabetes empowerment - Diabetes self-efficacy - Diabetes knowledge - Perceived stress - Depression - Satisfaction with life - Overall well-being | - This study suggests that, for young adults with a history of lapses in care, a structured transition program is effective in lowering blood glucose, reducing severe hypoglycemia and emergency department utilization, and improving uptake of routine diabetes care - Loss to follow-up and psychosocial concerns remain significant challenges in this population |
| Qayyum, Lone, Ibrahim, Atta, & Raza, 2010  Pakistan | Diabetes 1 | Longitudinal (3 months), control group: none | *Intervention:* diabetes self-management education  *Description:* covers pathology and information, insulin and glucose management, planning for special needs, sick days management, hypoglycemia management, physical activity, travelling and nutrition  *Mode:* group, with parents  *Personnel:* multidisciplinary pediatric diabetes team including an endocrinologist, general pediatrician, nutritionist and diabetic nurse  *Delivery method*: face to face with PowerPoint sessions, kid sack with equipment for self-care  *Duration:* two sessions of 1.5 hours, monthly revision exercises over 3 months.  *Age:* mean age 9.9 years  *Setting:* hospital: Diabetic Clinic of the National Institute of Child Health | - Body mass index - Insulin regime - Blood glucose | - Out of a total of 60 patients, 50 completed the trial - There was a significant decrease in the blood glucose levels after the intervention |
| Rathleff, Roos, Olesen, & Ras-mussen, 2015  Denmark | Patello-femoral pain | RCT (2 years), control group: patient education | *Intervention:* patient education and exercise therapy  *Description:* patient education covers pain management, how to modify physical activity using pacing and load management strategies, information on optimal knee alignment during daily tasks and responses to questions from the adolescent or the parents. The exercise therapy is an evidence-based combination of supervised group training sessions and unsupervised home-based exercises.  *Mode*: individual with parent  *Personnel:* physiotherapist  *Delivery method:* face to face and leaflet, home-based exercises  *Duration:* 30 minutes, 3 per week, 3 months  *Age:* 15-19, mean age 17.3 years  *Setting:* school | - Recovery - Quality of life - Physical activity - Sports participation - Knee injury and osteoarthritis outcome | - Adolescents randomized to patient education and exercise therapy were more likely to have recovered at 12 months - Similar results were observed at 3 and 6 months while the effect was further increased at 24 months - A higher total number of weekly exercise sessions increased the odds of recovery |
| Santiprabhob et al., 2014  Thailand | Obesity | Longitudinal (12 months), control group: none | *Intervention:* group-based treatment program  *Description:* information about the consequences of obesity and lifestyle modifications. Initial education in hospital, maintaining lifestyle in out-patient phase. Addressed obesity facts, the importance of being active and healthy lifestyle. Nutritional instructions were delivered, including how to choose healthy food, prepare healthy dishes at home, and count calories simply  *Mode:* group, family  Personnel: physicians, nurses, nutritionists, and medical staff  *Delivery method:* lecture, interactive discussion, and games  *Duration:* 2-3 hours, 5 sessions, 9 months  *Age:* mean age 12.3 years  *Setting:* hospital | - Weight control - Metabolic profiles - Obesity-related complications | - Their percentage weight for height and percentage body fat decreased significantly - Insulin resistance, lipid profiles, and transaminases levels improved - The prevalence of prediabetes, dyslipidemia, and elevated transaminases decreased significantly - The participants perceived the program as valuable |
| Schmidt, Herrmann-Garitz, Bomba, & Thyen, 2016  Germany | Diabetes 1, cystic fibrosis or inflammatory bowel disease | RCT (6 months), control group: ordinary care | *Intervention:* generic transition-oriented patient education program  *Description*: newly established generic patient education program employing transition workshops, standardized modules on adjustment to adult care settings, organization of future disease management, career choices and partnership. Covers transfer to adult medicine, orientation in the health system, future planning and occupation, separation from parents, communication about illness with peers and partners, stress management and activation of resources. Some modules include condition-specific aspects  *Mode:* group, workshops  Personnel: psychologist and pediatrician  *Delivery method:* face to face through various interactive group methods and learning processes  *Duration:* eight sessions of 60-90 minutes over 2 days  *Age:* mean age 16.8 years  *Setting:* pediatric outpatient clinics and rehabilitation centers with in-patients | - Health-related transition competence - Self-efficacy - Satisfaction with care - Patient activation - Quality of life | - The intervention significantly affected transition competence, self-efficacy and satisfaction with school care six months post intervention - The intervention did not significantly affect patient activation and quality of life - Post-hoc analysis suggested different effects across conditions |
| Sequeira et al., 2015  USA | Diabetes 1 | Longitudinal (12 months), control group: ordinary care | *Intervention:* Let’s Empower and Prepare  *Description*: theory based, structured transition program incorporating tailored diabetes education on diabetes basics, sick day management, use of alcohol and recreational drugs, contraception and family planning, and accessing care in the adult health care system, quarterly. Case managers facilitated program delivery, coordinated transfer from the pediatrics clinic to the adult clinic, and encouraged adherence to scheduled clinic visits  *Mode*: group  *Personnel:* multidisciplinary care team, including a certified diabetes educator and dietitian, registered dietitian, internal medicine-pediatrics-trained physician with fellowship in adult endocrinology  *Delivery method*: face to face with education classes, website  *Duration:* 12 months  *Age*: mean age 19.7 years  *Setting:* hospital, pediatric clinics | - Routine clinic visits - Glycemic control - Hypoglycemia - Health care use - Psychosocial well-being | - The intervention was successful in facilitating transition to adult care without a decrease in clinical follow-up - Compared with usual care, the intervention facilitated improvements in glycemic control and psychosocial well-being - No significant changes in diabetes empowerment, diabetes knowledge, perceived stress, or life satisfaction |
| Strom-back, Malmgren-Olsson, & Wiklund, 2013  Sweden | Self-defined stress-related problems | Qualitative, interviews | *Intervention*: stress management course  *Description*: combination of well-established and evidence-based methods. physiotherapeutic body-based, health-promoting, gender-sensitive stress management intervention. stress management course covers stress and pressures related to body ideals, body awareness and relaxation  *Mode:* group  *Personnel:* physiotherapist  Delivery method: reflective discussions; short general lectures, physiotherapeutic methods, logbook  *Duration*: eight sessions of 2 hours  *Age:* 16-25 years  *Setting*: youth centers | - Experiences from participating in the intervention | - The intervention can be said to represent a safe and explorative, individual and collective space, which facilitated gender identity work, social support, and confirmation - It also represents a bodily space, as well as a breathing space, that served to decrease stress symptoms and supported the discovery of bodily resources such as strength, stability, and relaxation |
| Tan & Martin, 2013  Australia | Mental health disorder | RCT (3 months), control group: ordinary care | *Intervention*: mindfulness-based intervention  *Description:* mindfulness training based on the adult MBSR protocol, but modified to suit adolescents’ developmental needs. Covers balance of present moment attention and acceptance with a non-judgmental openness to all experiences, regardless of their valence. Emotional release and affect regulation, mindful drawing, mindful eating, mindful listening to music and sculpting  *Mode:* group  *Personnel:*  *Delivery method*: face to face lectures, exercises, discussions, homework, handouts, reminder via mobile phone text messages  *Duration*: 5 weeks, five sessions of 10 minutes  *Age:* mean age 15.4 years  *Setting:* community mental health clinics | - Depression and anxiety stress - Self-esteem - Resiliency - Mastery - Relatedness - Emotional reactivity - Avoidance and fusion - Acceptance and mindfulness - Behavior | - The effect of treatment group on mental health change was reduced and became statistically non-significant when mindfulness was entered into the model, establishing that mindfulness change completely mediated the impact of treatment group on mental health change |
| Trollvik, Rings-berg, & Silen, 2013  Norway | Asthma | Qualitative | *Intervention:* asthma education  *Description:* based on asthma guidelines, developed by co-operative inquiry. Covers definition of asthma, asthma trigger factors, how it feels to have asthma, how to cope with asthma, the location of the lungs in the body, asthma medications, physical activity with respect to asthma and whether to be open about asthma or not  *Mode*: group, education, storytelling, pictures, storybook  *Personnel:* nurse specialized in asthma, physiotherapist  *Delivery method*: face to face  *Duration*: not reported  *Age:* 8 and 10 years reported  *Setting:* hospital | - Responses of children to the intervention | - The unique aspect about this intervention is that it emanates from children’s perspectives - The children were actively involved and learnt from each other’s shared knowledge and experiences, which is a good source of meaningful learning and empowering processes |
| Turkeli, Yilmaz, & Yuksel, 2016  Turkey | Asthma | Longitudinal (2 months), control group: none | *Intervention:* asthma education  *Description:* standardized education on proper MDI-spacer use and asthma control  *Mode:* individual, with parent, talk and demonstration  *Personnel:* pediatric allergy and pulmonology nurse  *Delivery method:* face to face with device demonstrations and education about use  *Duration:* 1 session  *Age:* 2.5-13, mean age 7.5 years  *Setting:* hospital | - Inhalation technique - Asthma control - Asthma symptoms | - Providing standardized education about metered dose inhaler with a spacer device use to children and parents leads to correct use and is associated with improvement in asthma symptom score and asthma control |
| van Bragt et al., 2015  Australia | Asthma | RCT (9 months), control group: ordinary care | *Intervention*: self-management support  *Description:* optimized to the needs of children. A specific problem selected through shared decision-making would be the subject of treatment, leading to a written action plan  *Mode:* individual with parent  *Personnel:* nurse-led  *Delivery method:* face to face  *Duration:*  *Age:* 6-11, mean age 8.6 years  *Setting:* general practices | - Asthma control - Quality of life - Strengths and difficulties - Subjective asthma experience assessed by two questions | - Treatment differences not significantly related to quality of life - Due to recruitment problems and underpowered analyses, no firm conclusions on the effectiveness could be drawn - Still, this can be considered a valuable pilot study. In the future, general practices may have better capacity to commit to such treatment |
| Velsor-Friedrich et al., 2012  USA | Asthma | RCT (12 months), control group: ordinary care | *Intervention:* coping skills training  *Description:* asthma education on asthma triggers, inhaler techniques, warning symptoms, medication adherence. Coping skills training (CST) on cognitive behavioral strategy, which is a component of the supportive-educative nursing system to be delivered to the students in the treatment group. The goal of CST is to teach children and adolescents personal and social coping skills that can assist them in dealing with potential stressors they encounter in their daily lives and the stress reactions that may result from these situations. Teaches such skills as social problem-solving, effective communication and social skills, managing stress, conflict resolution around issues associated with asthma, cognitive restructuring (guided self-dialogue)  *Mode*: group (and individual clinic visit)  *Personnel:* asthma education: nurse practitioner CST: doctoral student was trained by the PI to conduct the CST sessions. The training included five two-hour sessions  *Delivery method*: face to face education, gaming, role-play, problem-solving work  *Duration:* both: five education sessions, 1 clinical visit. Coping skills training: Five sessions of 45 minutes, over 6 weeks, with one makeup session, to make a total of six sessions. Booster CST session was offered at 2 months after completion of all of the CST sessions.  *Age:* 13-19, mean age 15.8 years  *Setting:* high school | - Self-care - Asthma quality of life - Knowledge about asthma - Asthma self-efficacy - Coping | - Both groups improved over time, with significant increases in asthma-related quality of life, asthma knowledge, and asthma self-efficacy, accompanied by decreases in symptom days and asthma-related school absences - Findings suggest that coping-skills training as implemented in this study provided no additional benefit beyond that experienced in the control group - However, group-based interventions delivered in the school setting may be beneficial for low-income, minority teens with asthma |
| Wang et al., 2010  USA | Diabetes 1 | RCT (9 months), control group: diabetes education | *Intervention:* motivational interviewing-based education and structured diabetes education  *Description:* both theory-based, the structured diabetes program used comprehensive checklists compiled using core content recommendations from the American Diabetes Association on medication, monitoring, acute complications, and lifestyle  *Mode:* individual  *Personnel:* diabetes educators, trained in motivational interviewing at two-day workshops  *Delivery method:* face to face  *Duration:* 9 months  *Age:* 12-18, mean age15 years  *Setting:* children’s medical center | - Blood glucose - Depression - Diabetes-related quality of life - Self-care | - Over the 6 months of follow-up, the intervention group given structured diabetes education had lower adjusted mean blood glucose value than the group given motivational interviewing-based education - There were no differences on any of the psychosocial measures |
| Watson et al., 2009  Canada | Asthma | RCT (12 months), control group: ordinary care | *Intervention*: interactive education  *Description*: small-group interactive program of education about asthma. Developed based on recommendations from the Canadian asthma consensus statement on education about asthma. Basic epidemiology and pathophysiology, goals of management, warning signs, triggers, avoidance, control strategies, asthma diary, medication use, demonstrations of devices and techniques, asthma action plan, self-management.  *Mode*: For children 3-6 years, parents were participants; for children 7-11 years there were separate groups children and parents; for children 12-16 the groups were adolescents only.  *Personnel:* nurse-educator experienced in asthma management, and respiratory therapist  *Delivery method:* face to face and booklet, e-mail  *Duration*: four sessions of 1.5 hour  *Age*: 3-16 years  *Setting:* hospital | - Emergency Department visits - Hospitalization - Quality of life - Use of oral corticosteroid therapy for exacerbations of asthma - Productivity in the workplace and pulmonary function | - During the year after enrolment, children in the intervention group made significantly fewer visits to the emergency department compared with those in the control group - The likelihood of a child in the intervention group requiring emergency care was reduced - Fewer courses of oral corticosteroids were required by children in the intervention group than by those in the control group - No impact on hospital admissions was observed |
| Wu et al., 2014  Taiwan | Cancer | Mixed methods: RCT (3 months), control group: ordinary care, and qualitative descriptions | *Intervention:* psycho-educational intervention  *Description*: theory based with manual containing the major characteristics with cancer experience distress related to illness and treatments. Modules to identify participants’ stressors related to treatment side effects during hospitalization and their self-help principles, to incorporate self-help methods and generate effective coping skills to solve participants’ problems and to provide participants with the opportunity to share their experience after engaging in positive coping.  *Mode:* group  *Personnel:* well-trained research assistant  *Delivery method:* face to face with sharing experience and feelings and coping exercises  *Duration:* three sessions of 60-90 minutes, 3 months follow-up with sharing experience, online game information, Facebook support  *Age:* mean age 13.2 years  *Setting:* hospital | - Perceived symptoms - Cancer coping - Satisfaction with program - Feelings about attending the intervention and what they had learned | - No significant difference in coping scores was found between groups, but the intervention group reported significantly lower scores in gastrointestinal problems and pain - Most symptoms decreased significantly over time in both groups, except for gastrointestinal problems - The scores in pain, bone marrow suppression and body image showed significant interaction effects between groups on changes over time - Qualitative results showed that participants evaluated the intervention positively, especially about receipt of psychological support and learnt coping skills |
| Zivkovic, Radic, Cerovic, & Vukasinovic, 2008  Serbia | Asthma | Longitudinal (12 months), control group: none | *Intervention*: Asthma education intervention, asthma school  *Description:* covers asthma basics, inhalation techniques, compliance, allergen avoidance, environmental control, sports  *Mode:* group with family.  *Personnel:* trained asthma educators including pediatric pulmonologists, respiratory nurses and social nurse specialist  *Delivery method:* face to face with lectures, audiovisual presentations, open discussions, workshops  *Duration*: 2 x half day.  *Age:* –5-18, mean age 11.5 years  *Setting:* hospital | - Asthma perception - Knowledge - Self-management | - The results confirm the effectiveness of educational intervention for childhood asthma - For the intervention group, there was improvement regarding the regular intake of anti-asthma therapy and inhalation technique after the education - The dosage of inhaled corticosteroids was reduced 12 months after the intervention - The percentage of parents with sufficient asthma knowledge score showed no significant change in the non-intervention group |

Differences or changes were considered significant at p≥0.05

Table A. Characteristics of interventions, outcomes and conclusions

Al-sheyab, N., Gallagher, R., Crisp, J., & Shah, S. (2012). Peer-led education for adolescents with asthma in Jordan: a cluster-randomized controlled trial. *Pediatrics, 129*(1), e106-112. doi:10.1542/peds.2011-0346

Almomani, B. A., Mayyas, R. K., Ekteish, F. A., Ayoub, A. M., Ababneh, M. A., & Alzoubi, S. A. (2017). The effectiveness of clinical pharmacist's intervention in improving asthma care in children and adolescents: Randomized controlled study in Jordan. *Patient Educ Couns, 100*(4), 728-735. doi:10.1016/j.pec.2016.11.002

Altundag, S., & Bayat, M. (2016). Peer Interaction and Group Education for Adaptation to Disease in Adolescents with Type 1 Diabetes Mellitus. *Pak J Med Sci, 32*(4), 1010-1014. doi:10.12669/pjms.324.9809

Arikan-Ayyildiz, Z., Isik, S., Caglayan-Sozmen, S., Anal, O., Karaman, O., & Uzuner, N. (2016). Efficacy of asthma education program on asthma control in children with uncontrolled asthma. *Turk J Pediatr, 58*(4), 383-388.

Bean, M. K., Powell, P., Quinoy, A., Ingersoll, K., Wickham, E. P., 3rd, & Mazzeo, S. E. (2015). Motivational interviewing targeting diet and physical activity improves adherence to paediatric obesity treatment: results from the MI Values randomized controlled trial. *Pediatr Obes, 10*(2), 118-125. doi:10.1111/j.2047-6310.2014.226.x

Bowen, F. (2013). Asthma education and health outcomes of children aged 8 to 12 years. *Clin Nurs Res, 22*(2), 172-185. doi:10.1177/1054773812461914

Bryant-Stephens, T., & Li, Y. (2008). Outcomes of a home-based environmental remediation for urban children with asthma. *J Natl Med Assoc, 100*(3), 306-316.

Burkhart, P. V., Rayens, M. K., Oakley, M. G., Abshire, D. A., & Zhang, M. (2007). Testing an intervention to promote children's adherence to asthma self-management. *J Nurs Scholarsh, 39*(2), 133-140. doi:10.1111/j.1547-5069.2007.00158.x

Butz, A. M., Halterman, J., Bellin, M., Kub, J., Tsoukleris, M., Frick, K. D., . . . Bollinger, M. E. (2014). Improving preventive care in high risk children with asthma: lessons learned. *J Asthma, 51*(5), 498-507. doi:10.3109/02770903.2014.892608

Cabrera, S. M., Srivastava, N. T., Behzadi, J. M., Pottorff, T. M., Dimeglio, L. A., & Walvoord, E. C. (2013). Long-term glycemic control as a result of initial education for children with new onset type 1 diabetes: does the setting matter? *Diabetes Educ, 39*(2), 187-194. doi:10.1177/0145721713475845

Campbell, J. D., Brooks, M., Hosokawa, P., Robinson, J., Song, L., & Krieger, J. (2015). Community Health Worker Home Visits for Medicaid-Enrolled Children With Asthma: Effects on Asthma Outcomes and Costs. *Am J Public Health, 105*(11), 2366-2372. doi:10.2105/AJPH.2015.302685

Cardoso Tde, A., Farias Cde, A., Mondin, T. C., da Silva Gdel, G., Souza, L. D., da Silva, R. A., . . . Jansen, K. (2014). Brief psychoeducation for bipolar disorder: impact on quality of life in young adults in a 6-month follow-up of a randomized controlled trial. *Psychiatry Res, 220*(3), 896-902. doi:10.1016/j.psychres.2014.09.013

Chalder, T., Deary, V., Husain, K., & Walwyn, R. (2010). Family-focused cognitive behaviour therapy versus psycho-education for chronic fatigue syndrome in 11- to 18-year-olds: a randomized controlled treatment trial. *Psychol Med, 40*(8), 1269-1279. doi:10.1017/S003329170999153X

Chiang, L. C., Ma, W. F., Huang, J. L., Tseng, L. F., & Hsueh, K. C. (2009). Effect of relaxation-breathing training on anxiety and asthma signs/symptoms of children with moderate-to-severe asthma: a randomized controlled trial. *Int J Nurs Stud, 46*(8), 1061-1070. doi:10.1016/j.ijnurstu.2009.01.013

Clark, N. M., Shah, S., Dodge, J. A., Thomas, L. J., Andridge, R. R., & Little, R. J. (2010). An evaluation of asthma interventions for preteen students. *J Sch Health, 80*(2), 80-87. doi:10.1111/j.1746-1561.2009.00469.x

Coughlin, C. C., Perez, M., Kumar, M. G., Jeffe, D. B., Bayliss, S. J., & Sternhell-Blackwell, K. (2017). Skin cancer risk education in pediatric solid organ transplant patients: an evaluation of knowledge, behavior, and perceptions over time. *Pediatr Transplant, 21*(2). doi:10.1111/petr.12817

Curtis, J., Watkins, A., Rosenbaum, S., Teasdale, S., Kalucy, M., Samaras, K., & Ward, P. B. (2016). Evaluating an individualized lifestyle and life skills intervention to prevent antipsychotic-induced weight gain in first-episode psychosis. *Early Interv Psychiatry, 10*(3), 267-276. doi:10.1111/eip.12230

Davis, A. M., Benson, M., Cooney, D., Spruell, B., & Orelian, J. (2011). A matched-cohort evaluation of a bedside asthma intervention for patients hospitalized at a large urban children's hospital. *J Urban Health, 88 Suppl 1*, 49-60. doi:10.1007/s11524-010-9517-6

Dingemann, J., Szczepanski, R., Ernst, G., Thyen, U., Ure, B., Goll, M., & Menrath, I. (2017). Transition of Patients with Esophageal Atresia to Adult Care: Results of a Transition-Specific Education Program. *Eur J Pediatr Surg, 27*(1), 61-67. doi:10.1055/s-0036-1587334

Espinoza-Palma, T., Zamorano, A., Arancibia, F., Bustos, M. F., Silva, M. J., Cardenas, C., . . . Prado, F. (2009). Effectiveness of asthma education with and without a self-management plan in hospitalized children. *J Asthma, 46*(9), 906-910. doi:10.3109/02770900903199979

Fincher, W., Shaw, J., & Ramelet, A. S. (2012). The effectiveness of a standardised preoperative preparation in reducing child and parent anxiety: a single-blind randomised controlled trial. *J Clin Nurs, 21*(7-8), 946-955. doi:10.1111/j.1365-2702.2011.03973.x

Flapper, B. C., Duiverman, E. J., Gerritsen, J., Postema, K., & van der Schans, C. P. (2008). Happiness to be gained in paediatric asthma care. *Eur Respir J, 32*(6), 1555-1562. doi:10.1183/09031936.00140407

Garanty-Bogacka, B., Syrenicz, M., Goral, J., Krupa, B., Syrenicz, J., Walczak, M., & Syrenicz, A. (2011). Changes in inflammatory biomarkers after successful lifestyle intervention in obese children. *Endokrynol Pol, 62*(6), 499-505.

Garvik, M., Idsoe, T., & Bru, E. (2014). Effectiveness study of a CBT-based adolescent coping with depression course. *Emotional and Behavioural Difficulties, 19*(2), 195-209.

Gordon, K., Murin, M., Baykaner, O., Roughan, L., Livermore-Hardy, V., Skuse, D., & Mandy, W. (2015). A randomised controlled trial of PEGASUS, a psychoeducational programme for young people with high-functioning autism spectrum disorder. *J Child Psychol Psychiatry, 56*(4), 468-476. doi:10.1111/jcpp.12304

Grey, M., Whittemore, R., Jaser, S., Ambrosino, J., Lindemann, E., Liberti, L., . . . Dziura, J. (2009). Effects of coping skills training in school-age children with type 1 diabetes. *Res Nurs Health, 32*(4), 405-418. doi:10.1002/nur.20336

Guner, U. C., & Celebioglu, A. (2015). Impact of symptom management training among asthmatic children and adolescents of self-efficacy and disease course. *Journal of Asthma, 52*(8), 858-865.

Haeberli, S., Grotzer, M. A., Niggli, F. K., Landolt, M. A., Linsenmeier, C., Ammann, R. A., & Bodmer, N. (2008). A psychoeducational intervention reduces the need for anesthesia during radiotherapy for young childhood cancer patients. *Radiat Oncol, 3*, 17. doi:10.1186/1748-717X-3-17

Hashemipour, M., Kelishadi, R., Tavalaee Zavvareh, S. A., & Ghatreh-Samani, S. (2012). Effect of education on anthropometric indices in obese parents and children after one year of follow-up. *ARYA Atheroscler, 8*(1), 21-26.

Herbert, L. J., Sweenie, R., Kelly, K. P., Holmes, C., & Streisand, R. (2014). Using Qualitative Methods to Evaluate a Family Behavioral Intervention for Type 1 Diabetes. *Journal of Pediatric Health Care, 28*(5), 376-385.

Holmes, C. S., Chen, R., Mackey, E., Grey, M., & Streisand, R. (2014). Randomized clinical trial of clinic-integrated, low-intensity treatment to prevent deterioration of disease care in adolescents with type 1 diabetes. *Diabetes Care, 37*(6), 1535-1543. doi:10.2337/dc13-1053

Horner, S. D., & Fouladi, R. T. (2008). Improvement of rural children's asthma self-management by lay health educators. *J Sch Health, 78*(9), 506-513. doi:10.1111/j.1746-1561.2008.00336.x

Indinnimeo, L., Bonci, E., Capra, L., La Grutta, S., Monaco, F., Paravati, F., . . . Duse, M. (2009). Clinical effects of a Long-term Educational Program for children with asthma - Aironet. A 1-yr randomized controlled trial. *Pediatr Allergy Immunol, 20*(7), 654-659. doi:10.1111/j.1399-3038.2009.00857.x

Janssens, T., & Harver, A. (2015). Effects of Symptom Perception Interventions on Trigger Identification and Quality of Life in Children with Asthma. *Pulm Med, 2015*, 825137. doi:10.1155/2015/825137

Julian, V., Amat, F., Petit, I., Pereira, B., Fauquert, J. L., Heraud, M. C., . . . Labbe, A. (2014). Impact of a short early therapeutic education program on the quality of life of asthmatic children and their families. *Pediatr Pulmonol*. doi:10.1002/ppul.23013

Katz, M. L., Volkening, L. K., Butler, D. A., Anderson, B. J., & Laffel, L. M. (2014). Family-based psychoeducation and Care Ambassador intervention to improve glycemic control in youth with type 1 diabetes: a randomized trial. *Pediatr Diabetes, 15*(2), 142-150. doi:10.1111/pedi.12065

Kelsey, M. M., Geffner, M. E., Guandalini, C., Pyle, L., Tamborlane, W. V., Zeitler, P. S., . . . Youth Study, G. (2016). Presentation and effectiveness of early treatment of type 2 diabetes in youth: lessons from the TODAY study. *Pediatr Diabetes, 17*(3), 212-221. doi:10.1111/pedi.12264

Kenney, A., Chambers, R. A., Rosenstock, S., Neault, N., Richards, J., Reid, R., . . . Barlow, A. (2016). The Impact of a Home-Based Diabetes Prevention and Management Program on High-Risk American Indian Youth. *Diabetes Educ, 42*(5), 585-595. doi:10.1177/0145721716658357

Krieger, J., Takaro, T. K., Song, L., Beaudet, N., & Edwards, K. (2009). A randomized controlled trial of asthma self-management support comparing clinic-based nurses and in-home community health workers: the Seattle-King County Healthy Homes II Project. *Arch Pediatr Adolesc Med, 163*(2), 141-149. doi:10.1001/archpediatrics.2008.532

Laguna-Alcaraz, A. D., Mejia-Rodriguez, O., Rendon-Paredes, A. L., Villa-Barajas, R., & Paniagua, R. (2017). Impact of a comprehensive intervention to families with teenage sons with overweight and obesity in a primary care setting: A case report. *Diabetes Metab Syndr, 11 Suppl 1*, S195-S200. doi:10.1016/j.dsx.2016.12.031

Larson, A., Ward, J., Ross, L., Whyatt, D., Weatherston, M., & Landau, L. (2010). Impact of structured education and self management on rural asthma outcomes. *Aust Fam Physician, 39*(3), 141-144.

Li, H. C., Chung, O. K., Ho, K. Y., Chiu, S. Y., & Lopez, V. (2013). Effectiveness of an integrated adventure-based training and health education program in promoting regular physical activity among childhood cancer survivors. *Psycho-Oncology, 22*(11), 2601-2610. doi:10.1002/pon.3326

Lloyd, S., Chalder, T., & Rimes, K. A. (2012). Family-focused cognitive behaviour therapy versus psycho-education for adolescents with chronic fatigue syndrome: long-term follow-up of an RCT. *Behav Res Ther, 50*(11), 719-725. doi:10.1016/j.brat.2012.08.005

Magzamen, S., Patel, B., Davis, A., Edelstein, J., & Tager, I. B. (2008). Kickin' Asthma: school-based asthma education in an urban community. *J Sch Health, 78*(12), 655-665. doi:10.1111/j.1746-1561.2008.00362.x

Majumdar, I., Bethin, K., & Quattrin, T. (2015). Weight trajectory of youth with new-onset type 1 diabetes comparing standard and enhanced dietary education. *Endocrine, 49*(1), 155-162. doi:10.1007/s12020-014-0469-6

Maslow, G., Adams, C., Willis, M., Neukirch, J., Herts, K., Froehlich, W., . . . Rickerby, M. (2013). An evaluation of a positive youth development program for adolescents with chronic illness. *J Adolesc Health, 52*(2), 179-185. doi:10.1016/j.jadohealth.2012.06.020

McGhan, S. L., Wong, E., Sharpe, H. M., Hessel, P. A., Mandhane, P., Boechler, V. L., . . . Befus, A. D. (2010). A children's asthma education program: Roaring Adventures of Puff (RAP), improves quality of life. *Can Respir J, 17*(2), 67-73.

Melnyk, B. M., Jacobson, D., Kelly, S. A., Belyea, M. J., Shaibi, G. Q., Small, L., . . . Marsiglia, F. F. (2015). Twelve-Month Effects of the COPE Healthy Lifestyles TEEN Program on Overweight and Depressive Symptoms in High School Adolescents. *J Sch Health, 85*(12), 861-870. doi:10.1111/josh.12342

Murphy, H. R., Wadham, C., Hassler-Hurst, J., Rayman, G., Skinner, T. C., Families, . . . Teamwork Study, G. (2012). Randomized trial of a diabetes self-management education and family teamwork intervention in adolescents with Type 1 diabetes. *Diabet Med, 29*(8), e249-254. doi:10.1111/j.1464-5491.2012.03683.x

Ng, S. M., Li, A. M., Lou, V. W., Tso, I. F., Wan, P. Y., & Chan, D. F. (2008). Incorporating family therapy into asthma group intervention: a randomized waitlist-controlled trial. *Fam Process, 47*(1), 115-130.

Otsuki, M., Eakin, M. N., Rand, C. S., Butz, A. M., Hsu, V. D., Zuckerman, I. H., . . . Riekert, K. A. (2009). Adherence feedback to improve asthma outcomes among inner-city children: a randomized trial. *Pediatrics, 124*(6), 1513-1521. doi:10.1542/peds.2008-2961

Powers, S. W., Kashikar-Zuck, S. M., Allen, J. R., LeCates, S. L., Slater, S. K., Zafar, M., . . . Hershey, A. D. (2013). Cognitive behavioral therapy plus amitriptyline for chronic migraine in children and adolescents: a randomized clinical trial. *JAMA, 310*(24), 2622-2630. doi:10.1001/jama.2013.282533

Price, K. J., Knowles, J. A., Fox, M., Wales, J. K., Heller, S., Eiser, C., . . . group, K. I.-O. S. (2016). Effectiveness of the Kids in Control of Food (KICk-OFF) structured education course for 11-16 year olds with Type 1 diabetes. *Diabet Med, 33*(2), 192-203. doi:10.1111/dme.12881

Pyatak, E. A., Sequeira, P. A., Vigen, C. L., Weigensberg, M. J., Wood, J. R., Montoya, L., . . . Peters, A. L. (2017). Clinical and Psychosocial Outcomes of a Structured Transition Program Among Young Adults With Type 1 Diabetes. *J Adolesc Health, 60*(2), 212-218. doi:10.1016/j.jadohealth.2016.09.004

Qayyum, A. A., Lone, S. W., Ibrahim, M. N., Atta, I., & Raza, J. (2010). Effects of diabetes self-management education on glycaemic control in children with insulin-dependent diabetes mellitus. *J Coll Physicians Surg Pak, 20*(12), 802-805. doi:12.2010/JCPSP.802805

Rathleff, M. S., Roos, E. M., Olesen, J. L., & Rasmussen, S. (2015). Exercise during school hours when added to patient education improves outcome for 2 years in adolescent patellofemoral pain: a cluster randomised trial. *Br J Sports Med, 49*(6), 406-412. doi:10.1136/bjsports-2014-093929

Santiprabhob, J., Leewanun, C., Limprayoon, K., Kiattisakthavee, P., Wongarn, R., Aanpreung, P., & Likitmaskul, S. (2014). Outcomes of group-based treatment program with parental involvement for the management of childhood and adolescent obesity. *Patient Educ Couns, 97*(1), 67-74. doi:10.1016/j.pec.2014.07.002

Schmidt, S., Herrmann-Garitz, C., Bomba, F., & Thyen, U. (2016). A multicenter prospective quasi-experimental study on the impact of a transition-oriented generic patient education program on health service participation and quality of life in adolescents and young adults. *Patient Educ Couns, 99*(3), 421-428. doi:10.1016/j.pec.2015.10.024

Sequeira, P. A., Pyatak, E. A., Weigensberg, M. J., Vigen, C. P., Wood, J. R., Ruelas, V., . . . Peters, A. L. (2015). Let's Empower and Prepare (LEAP): Evaluation of a Structured Transition Program for Young Adults With Type 1 Diabetes. *Diabetes Care, 38*(8), 1412-1419. doi:10.2337/dc14-2577

Stromback, M., Malmgren-Olsson, E. B., & Wiklund, M. (2013). 'Girls need to strengthen each other as a group': experiences from a gender-sensitive stress management intervention by youth-friendly Swedish health services--a qualitative study. *BMC Public Health, 13*, 907. doi:10.1186/1471-2458-13-907

Tan, L., & Martin, G. (2013). Taming the adolescent mind: preliminary report of a mindfulness-based psychological intervention for adolescents with clinical heterogeneous mental health diagnoses. *Clin Child Psychol Psychiatry, 18*(2), 300-312. doi:10.1177/1359104512455182

Trollvik, A., Ringsberg, K. C., & Silen, C. (2013). Children's experiences of a participation approach to asthma education. *J Clin Nurs, 22*(7-8), 996-1004. doi:10.1111/jocn.12069

Turkeli, A., Yilmaz, O., & Yuksel, H. (2016). Metered dose inhaler-spacer use education effects on achieve asthma control in children. *Tuberk Toraks, 64*(2), 105-111.

van Bragt, S., van den Bemt, L., Kievits, R., Merkus, P., van Weel, C., & Schermer, T. (2015). PELICAN: a cluster-randomized controlled trial in Dutch general practices to assess a self-management support intervention based on individual goals for children with asthma. *J Asthma, 52*(2), 211-219. doi:10.3109/02770903.2014.952439

Velsor-Friedrich, B., Militello, L. K., Richards, M. H., Harrison, P. R., Gross, I. M., Romero, E., & Bryant, F. B. (2012). Effects of coping-skills training in low-income urban African-American adolescents with asthma. *J Asthma, 49*(4), 372-379. doi:10.3109/02770903.2012.660296

Wang, Y. C., Stewart, S. M., Mackenzie, M., Nakonezny, P. A., Edwards, D., & White, P. C. (2010). A randomized controlled trial comparing motivational interviewing in education to structured diabetes education in teens with type 1 diabetes. *Diabetes Care, 33*(8), 1741-1743. doi:10.2337/dc10-0019

Watson, W. T., Gillespie, C., Thomas, N., Filuk, S. E., McColm, J., Piwniuk, M. P., & Becker, A. B. (2009). Small-group, interactive education and the effect on asthma control by children and their families. *CMAJ, 181*(5), 257-263. doi:10.1503/cmaj.080947

Wu, L. M., Chiou, S. S., Sheen, J. M., Lin, P. C., Liao, Y. M., Chen, H. M., & Hsiao, C. C. (2014). Evaluating the acceptability and efficacy of a psycho-educational intervention for coping and symptom management by children with cancer: a randomized controlled study. *J Adv Nurs, 70*(7), 1653-1662. doi:10.1111/jan.12328

Zivkovic, Z., Radic, S., Cerovic, S., & Vukasinovic, Z. (2008). Asthma School Program in children and their parents. *World J Pediatr, 4*(4), 267-273. doi:10.1007/s12519-008-0049-z
